# Identification of Disease-Related Genes That Are Common between Alzheimer’s and Cardiovascular Disease Using Blood Genome-Wide Transcriptome Analysis

**DOI:** 10.3390/biomedicines9111525

**Published:** 2021-10-23

**Authors:** Taesic Lee, Hyunju Lee, the Alzheimer’s Disease Neuroimaging Initiative

**Affiliations:** 1 Department of Biomedical Science and Engineering, Gwangju Institute of Science and Technology, Gwangju 61005, Korea; ddasic123@gist.ac.kr; 2 Department of Family Medicine, Yonsei University Wonju College of Medicine, Wonju 26426, Korea; 3 School of Electrical Engineering and Computer Science, Gwangju Institute of Science and Technology, Gwangju 61005, Korea

**Keywords:** Alzheimer’s disease, cardiovascular disease, blood gene expression, disease-related molecular signatures, differential expression analysis, gene regulatory network, convergent functional genomics, DigSee

## Abstract

Accumulating evidence has suggested a shared pathophysiology between Alzheimer’s disease (AD) and cardiovascular disease (CVD). Based on genome-wide transcriptomes, specifically those of blood samples, we identify the shared disease-related signatures between AD and CVD. In addition to gene expressions in blood, the following prior knowledge were utilized to identify several candidate disease-related gene (DRG) sets: protein–protein interactions, transcription factors, disease–gene relationship databases, and single nucleotide polymorphisms. We selected the respective DRG sets for AD and CVD that show a high accuracy for disease prediction in bulk and single-cell gene expression datasets. Then, gene regulatory networks (GRNs) were constructed from each of the AD and CVD DRG sets to identify the upstream regulating genes. Using the GRNs, we identified two common upstream genes (GPBP1 and SETDB2) between the AD and CVD GRNs. In summary, this study has identified the potential AD- and CVD-related genes and common hub genes between these sets, which may help to elucidate the shared mechanisms between these two diseases.

## 1. Introduction

In 2018, approximately 50 million individuals were estimated to have dementia, which is expected to increase to 152 million by 2050 [1]. Alzheimer’s disease (AD) and vascular dementia (VaD) account for 50–75% [2,3] and 15–20% [3,4] of all dementia cases, respectively. Mixed dementia, which has the characteristics of both AD and VaD, is one of the main issues for dementia [5,6]. Approximately 10% of all dementia cases in Asia, Africa, and Latin America are considered mixed dementia [5].

Numerous studies have reported an association between dementia and CVD [7,8,9]. The Rotterdam study reported that a previous diagnosis of stroke or myocardial infarction (MI) was related to a decline in cognitive function [7]. The AgeCoDe study in Germany reported that patients with coronary heart disease experienced a greater decline in cognitive function [8]. For the link between AD and CVD, the Cardiovascular Heart Study (CHS) cohort in the US reported that a previous diagnosis of atherosclerotic diseases, such as CVD and peripheral artery disease, were significantly or potentially related to an increased risk of all-cause dementia, mixed dementia, and pure AD [9].

Several mechanisms are shared among AD and CVD, including cerebral hypoperfusion, micro-infarcts, micro-bleeding, low amyloid beta clearance, high amyloid beta production, and low soluble amyloid precursor proteins [10]. To elucidate the pathophysiological link between AD and CVD, Beeri et al. [11] reported that subjects with an *APOE4* allele had a stronger correlation between AD neuropathology (neurofibrillary tangle and neuritic plaque) and CVD than those without the *APOE4* allele.

Nho et al. [12] performed a genome-wide transcriptome meta-analysis using AD blood datasets, including those from the Alzheimer’s Disease Neuroimaging Initiative (ADNI), AddNeuroMed, and Mayo Clinic Study of Aging (MCSA), and identified five blood late-onset AD-related genes. Maciejak et al. [13] analyzed the whole gene expression patterns of the peripheral blood mononuclear cells obtained from MI patients and proposed several potential prognostic biomarkers associated with the progression of heart failure. However, a whole transcriptomic analysis to uncover the shared pathophysiology between AD and CVD has not yet been conducted.

Therefore, this study aimed to identify the common disease-associated signatures, specifically for the blood transcriptome, between AD and CVD based on the following four steps:

(1) The curation of blood candidate set of disease-related genes (DRGs);
(2) The selection of DRG sets with high prediction performance;
(3) The selection of DRG sets having convergent results with single-cell RNA seq-based findings;
(4) The identification of upstream genes via network analysis.

In previous studies, the molecular signatures associated with disease status or other phenotypes have been validated by comparing them with results obtained from multiple random sample [14] or random feature sets [15,16]. We performed both random sample and feature sets to validate the candidate DRGs. Several studies have reported that even gene sets randomly selected have significantly high disease predictive power [17] and may be useful to identify sub-classes [18]. Therefore, specific sets of DRGs were selected in this study using the following assumption: if a preliminary set of genes selected using statistical and domain knowledge-based methods could outperform randomly selected genes for disease prediction, then this gene set may contain DRGs.

## 2. Methods

**Figure 1:**
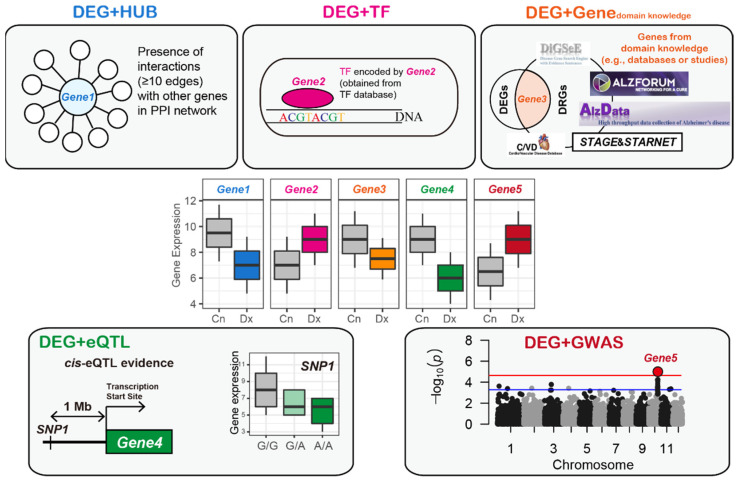
Selection of the preliminary sets of blood AD- and CVD-related genes. First, DEGs between different disease status (e.g., disease and healthy control) were selected using limma [19], following which the domain knowledge obtained from the disease–gene relationship databases was implemented to select the disease-related genes. DEG, differentially expressed gene; Cn, control; Dx, disease; PPI, protein–protein interaction; TF, transcription factor; eQTL, expression quantitative trait loci; GWAS, genome-wide association study.

The four steps taken in this study are as follows: First, disease-related genes were selected from blood gene expression datasets using statistical methods, a protein–protein interaction (PPI) network, TF database, disease–gene relationship database, blood cis-expression quantitative trait loci (eQTL) evidence, and disease-specific GWAS results (Figure 1). Then, a set of blood DRGs was selected if it improved the classification performance for the other tissue and same disease cases and the same tissue (blood) and other disease cases compared to a matched, randomly selected set of genes. Then, we selected the blood AD and CVD DRGs that were significantly conserved in the single-cell-based disease-associated signatures. Finally, gene regulatory networks (GRNs) were constructed from each AD and CVD DRG set to identify the upstream regulating genes and common upstream genes. All tasks, including the statistical analysis, establishment of prediction model, and measurement of prediction accuracy, were conducted using the R language (version 4.0.1).

### 2.1. Retrieval of Blood, Brain, Heart, Fat, and Vessel Transcriptomic Datasets

We retrieved six blood AD datasets (GSE63060, GSE63061, GSE85426, GSE4229, ADNI, and ROSMAP [20,21]), six brain AD datasets (GSE33000, GSE84422-GPL96, GSE84422-GPL97, GSE118553, GSE132903, and GSE5281), 11 blood CVD datasets (GSE60993, GSE20681, GSE59867, GSE90074, GSE34198, GSE12288, GSE20680, GSE9820, GSE62646, GSE66360, GSE7638), three heart CVD datasets (GSE57338, GSE1869, and GSE5406), one fat CVD dataset (GSE64554), and one carotid plaque CVD dataset (GSE43292). Most datasets were obtained from the Gene Expression Omnibus database [22]. The preprocessing procedure for each gene expression dataset is summarized in the Supplementary Materials.

### 2.2. Selection of High Quality Datasets for Feature Selection

A previous study [23] suggested that analyzing numerous gene expression datasets could cause a considerable amount of transcript information (due to the different platforms used for measuring gene expression) and trait-related signature data (due to the different phenotypical or disease statuses among the individual datasets) loss. Therefore, a MetaQC method that provides a score for the quality of the gene expression datasets for the meta-analysis [24] was used to enable the selection of high-quality blood gene expression datasets, which were then used for feature selection. However, running the MetaQC algorithm with numerous datasets simultaneously may cause a substantial loss of transcripts or probes due to the different platforms used and this means that only local checks can be made for the quality of a dataset using small remnant transcripts. To overcome this limitation, the quality indices were measured by iteratively selecting the *k* datasets from the *n* blood gene expression datasets, resulting in a blood dataset with _n__−1_C_k−1_ or cases including the blood dataset among _n_C_k_ for each quality index. The quality of the results for the blood dataset were determined by averaging the _n−1_C_k−1_ values for each quality index. Before checking the quality, the batch effect was iteratively adjusted in each loop of the running MetaQC among the selected *k* datasets using the ComBat method from the sva package (version 3.34.0) in the R language [25,26].

Pathway information is required to run the MetaQC algorithm. The Kyoto Encyclopedia of Genes and Genomes (KEGG) [27] database was obtained from MSigDB [28]. The results of the MetaQC include six quality indices (internal quality control (IQC), external QC (EQC), two accuracy QC indices (AQCg and AQCp), and two consistency QC indices (CQCg and CQCp)), and their averages formed the index, called the standardized mean rank (SMR) for each comparison of the gene expression datasets [24].

### 2.3. Differential Gene Expression Analysis

Differential gene expression analysis between two or three conditions was conducted using the “lmFit” and “eBayes” functions in the limma package (version 3.42.2) [19]. The results from the limma package included the fold-change (FC) values between the two statuses and the *p*-values for each gene. Genes with a false discovery rate (FDR)-adjusted *p*-value < 0.05 were defined as differentially expressed genes (DEGs).

### 2.4. Identification of Blood AD-Related Genes

The three blood AD datasets that had the top three SMR values in the MetaQC were selected. Subsequently, the batch effect among the three datasets was removed using the ComBat method [25] and the three datasets were integrated into a large blood AD dataset. From the large blood dataset, we identified DEGs with an FDR-adjusted *p*-value < 0.05 between AD and CN_AD_ (DEG_AD_).

A PPI network was compiled from the STRING database, which collected and integrated numerous interactions between the expressed proteins by consolidating the known and predicted PPIs from numerous studies [29]. The PPI network consisted of 11,759,454 edges among 38,708 proteins (based on the Ensembl Protein (ESPN)). A previous study reported that genes with 10 or more interactions in the PPI network generated accurate results for the prediction of blood AD [30]. Motivated by this study [30], the DEG_AD_ was mapped onto the PPI network [29] and identified the genes with ≥ 10 edges as DEG + HUB_AD_ (Figure 1).

Hägg et al. [31] collected genes known to be involved in transcription activities obtained from the GO database [32], yielding *LDB2* as an upstream gene related to atherosclerosis development. A previous study used the TFs obtained from TRANSFACT [33] to predict blood AD cases among the different cohorts [30]. In addition, the updated TF catalog by Lambert et al. [34] was used to identify the shared upstream blood genes between AD and diabetes [23]. Based on these studies, we implemented the TF list that had been manually updated by Lambert et al. [34]. Information on the TF-related genes was obtained from the Human Transcription Factors database, which is available at http://humantfs.ccbr.utoronto.ca/ [34]. There are two categories of TF-related genes in the database [34]: 1639 genes (known and likely human TFs) and 2765 genes (1639 TFs plus potential human TFs). The full list of 2765 TFs were used as these genes were included in one or more of the six TF databases or studies [34]. By conducting an intersection assessment between the DEG_AD_ and the 2765 TF-related genes [34], candidate DRGs were selected and annotated as DEG + TF_AD_ (Figure 1).

We used the DigSee search engine that identified gene–disease relationships using the text-mining method, and thus compiled a list of DRGs [35]. Using an “Alzheimer’s disease” query, approximately 2000 AD-related genes were obtained. Among them, we manually removed genes with probable false-positive results, yielding 1591 AD-related genes. Among DEG_AD_, AD-related genes from DigSee were defined as DEG + DIGSEE_AD_.

The AlzGene database comprehensively evaluates most genetic association studies in the field of AD [36] and is publicly available at http://alzgene.org/ (accessed on 5 September 2018). From this database, we manually extracted a list of 614 AD-related genes. The common genes between the DEG_AD_ and the 614 genes were defined as DEG + AlzGene.

Convergent functional genomics (CFG), a method for curating disease-related genes by integrating multiple lines of biological evidence obtained from human and animal models, has been used previously in the research of several diseases, such as psychotic and neurodegenerative diseases [30,37]. Specifically, Xu et al. [37] constructed an AD-CFG database to score all genes using five criteria, in which, if a gene satisfied the *k* criteria, the gene was scored as *k* (maximum score: 5). We manually scored DEG_AD_ using this database, which is publicly available at http://alzdata.org/ (accessed on 15 December 2020). Among them, genes with a CFG score ≥ 3 were selected and these genes were arranged as DEG + CFG.

Data in the form of summary statistics for blood cis-eQTL was obtained from a study that performed a blood whole-genome eQTL meta-analysis of 5311 samples from seven cohorts [38]. We selected gene single nucleotide polymorphism (SNP) pairs with an FDR-adjusted *p*-value < 0.05 for the cis-association between expression of the gene and types of the variant (eSNP), yielding 664,097 pairs that accounted for 5647 genes (annotated by Entrez ID). Among the 5647 genes with the blood cis-eQTL evidence [38], genes that were also DEGs were assigned as DEG + eQTL_AD_ (Figure 1).

The results in the form of summary statistics were downloaded from a GWAS meta-analysis of 17,008 patients with AD and 37,154 CNs obtained from four consortia [39]. We selected 15,422 SNPs with uncorrected *p*-values < 0.001 for the association between genetic variants and the presence of AD. The 15,422 SNPs were assigned to their corresponding genes using ANNOVAR [40] based on the RefSeq hg19 reference genome, yielding 2242 unique genes. Among the genes with GWAS evidence, those that were also DEG_AD_ were arranged as DEG + GWAS_AD_ (Figure 1).

### 2.5. Identification of Blood CVD-Related Genes

DEGs were identified using their recorded statuses for three diseases, including acute coronary syndrome (ACS), stable coronary artery disease (CAD), and CN, with the limma package (version 3.42.2). We defined genes with an FDR-adjusted *p*-value < 0.05 as DEG_CVD_.

In line with the process of selecting the blood AD-related genes (Section 2.4), DEG_CVD_ with ≥ 10 edges in the PPI network was defined as DEG + HUB_CVD_. Moreover, common genes between the DEG_CVD_ and 2765 TF-related genes were defined as DEG + TF_CVD_ and common genes between DEG_CVD_ with blood cis-eQTL evidence [38] were defined as DEG + eQTL_CVD_.

Using DigSee [35], the candidate CVD-related genes with a query of “Coronary Artery Disease” were compiled. To reduce false-positive results, a cut-off of 0.5 for the “EVIDENCE SENTENCE SCORE” was set, which yielded 1922 CVD-related genes. The DEGs identified in the CVD sample as well as one of the 1922 CVD-related genes, were defined as DEG + DIGSEE_CVD_.

Fernandes et al. [41] established C/VDdb, which is a CVD-related gene database constructed using a system-level integrative analysis for numerous CAD-associated studies. We manually extracted a list of 3495 CVD-related genes from the C/VDdb and the common genes between the DEG_CVD_ and the 3495 genes were defined as DEG + C/VDdb.

Talukdar et al. [42] applied a weighted gene co-expression network analysis [43] for seven tissues obtained from patients with late-stage CVD from the Stockholm Atherosclerosis Gene Expression (STAGE) study, yielding 171 modules (94 tissue-specific and 77 cross-tissue modules). Zeng et al. [44] applied STAGE results to seven tissues from the CVD patients enrolled in the Stockholm-Tartu Atherosclerosis Reverse Network Engineering Task (STARNET) study, yielding 98 modules to be replicated in STARNET. In addition, they selected the top 28 modules based on the degree of CAD heritability [44]. In total, 2943 multi-tissue CVD-related genes (mtCVD) were compiled from the 28 modules obtained from the seven tissues using STAGE and STARNET, and the common genes between the DEG_CVD_ and mtCVD were defined as DEG + mtCVD.

The statistical result was downloaded from the study performing a GWAS meta-analysis of 60,801 CAD patients and 123,504 CNs obtained from 48 studies in the form of summary statistics [45]. Furthermore, we selected 32,508 SNPs with uncorrected *p*-values < 0.001 for the association between genetic variants and the presence of CAD, and matched the 32,508 SNPs to their corresponding genes using ANNOVAR [40] based on the RefSeq hg19 reference genome, and this yielded 3245 unique genes. DEG_CVD_ with GWAS evidence were defined as DEG + GWAS_CVD_.

### 2.6. Evaluation of the Blood DRGs Based on Disease Classification Performance

The disease prediction performance of the selected DRGs was assessed based on the random sampling perspective. In detail, a random sample-sets pair was made by randomly assigning all samples in a gene expression dataset with a 0.7/0.3 ratio into train and test sets, respectively. Subsequently, we curated a random gene set that was matched with one of the candidate gene sets of the DRGs. The default setting of the support vector machine was used for the classification model. We iterated the disease prediction using the random sample-sets pair, the candidate gene sets of the DRGs, and the random gene set, yielding 1000 prediction performances for each of the candidate and random gene sets in the form of area under the receiver operating characteristic curves (AUCs). Note that a new random gene set was generated for each repetition, and samples randomly assigned in each repetition for training and testing model were the same between the candidates (e.g., blood AD- or CVD-related gene sets) and the random gene set. We compared 1000 pairs of the AUCs from the candidate DRGs and randomly selected genes based on a paired *t*-test. Detailed methods (e.g., classification model and sampling schemes) are described in the Supplementary Materials.

### 2.7. Comparison of the DRGs Obtained from the Blood and Single-Cell Datasets

The blood DRGs obtained from the blood bulk transcriptomic datasets were compared with the tissue DRGs obtained from the brain or heart single-cell analyses. For the single-cell analysis of the brain AD samples, we downloaded summary statistics of a single-cell RNA sequencing study (Supplementary Table S2 in this study [46]). In this study [46], samples (or cells) were classified into one of two statuses: “no pathology” or “AD pathology”. Those classified as “AD pathology” were further categorized into one of two groups: “early AD pathology” or “late AD pathology” based on nine clinicopathological traits [46]. The downloaded result in the form of a table consisted of three lists of FC values for all genes between the two conditions, including comparisons of “no pathology vs. AD pathology”, “no pathology vs. early AD pathology”, and “early AD pathology vs. late AD pathology” for each of the six cell types. Therefore, we curated 18 lists for the DEGs between the two conditions with an FDR-adjusted *p*-value < 0.05, from the brain single-cell study.

For the heart CVD single-cell analyses, a gene expression dataset was obtained from a study by Farbehi et al. [47]. This study performed single-cell RNA sequencing twice: first for the total interstitial cell types (TIP) and then for the Pdgfra-GFP^+^ fibroblast lineage cells (GFP). The preprocessing procedure for the heart single-cell dataset is summarized in the D Materials. For each of the cell types, we selected DEGs between the CVD (i.e., heart cells from MI-operated mice) and control (i.e., heart cells from sham-operated mice) with an FDR-adjusted *p*-value < 0.05, as cell type-specific CVD-related genes. The degree of enrichment of the cell-type specific DRGs with AD- and CVD-related gene sets (e.g., DEG + CFG, DEG + GWAS, and DEG + HUB) was measured using Fisher’s exact test.

### 2.8. Pathway Analysis

The degree of enrichment between the blood DRGs and the genes in specific pathways was measured using a hypergeometric test. Considering the gene set and pathway, the *p*-values were computed as in Equation (1):

(1) $$P-\mathrm{value}=\overset{\min\left(M,n\right)}{\underset{k=m}{\sum }}\frac{\left(\begin{array}{c} M\\ k \end{array}\right)\left(\begin{array}{c} N-M\\ n-k \end{array}\right)}{\left(\begin{array}{c} N\\ n \end{array}\right)}$$

where *N* represents the total number of genes in the gene expression dataset, *M* represents the number of genes in the pathway, *n* represents the number of genes in the gene set (i.e., DRGs), and *m* represents the number of genes that are common between the candidate DRGs and genes in the pathway. Pathways with an FDR-corrected *p*-value < 0.05 were defined as significantly enriched pathways. From MSigDB [9], the pathway information, including KEGG [8] and Gene Ontology [21], were obtained.

### 2.9. Establishment of a Gene Regulatory Network

A GRN was constructed using GENIE3, a GRN inference algorithm with a tree-based ensemble method [48]. A gene expression matrix (named as “exprMatrix” in the GENIE3 algorithm) and a list of candidate upstream genes (named as “regulator”) are needed to run GENIE3. GENIE3 generates a result table consisting of interactions between two genes and their weights. The weight is the degree of variable importance measured by summing the total variance reductions, indicating that a large weight value between two genes corresponds to actual interaction. An important issue for applying GRN is to estimate a regulatory direction of interaction between two genes. Zhang et al. [49] used genes with brain cis-expression (e)SNPs as anchors to establish a causal relationship between the genes in the AD-gene regulatory network (GRN). Zeng et al. [44] inferred a CVD GRN using genes with cis-eSNPs and TFs as priors to impose a direction between the genes. These two methods were integrated to construct a GRN with direction. Information about the TFs and blood eSNPs was obtained from the TF database [34] and the summary statistics of a previous study [38], respectively. Similar to the method by Zeng et al. [44], the TFs were determined as prior (named as “regulators”). The following four steps were applied to make the edge deletions and selections:

(1) To reduce the false-positive edges, we selected edges between the genes with weight values in which the degree of interaction strength calculated by GENIE3 was greater than the mean plus two standard deviations of the weight values.
(2) Similar to a study by Zhang et al. [49], we excluded cases (i.e., interactions or edges) in which the genes without any cis-eSNPs were parents of genes with one or more cis-eSNPs. There were some cases in which the parent and child genes both had cis-eSNPs, which is referred to as bi-directional edges. Kirsten et al. [50] suggested that genes are not only regulated by the most significant cis-eSNP but also by a considerable number of other possible cis-regulations. Jansen et al. [51] hypothesized that a cis-eSNP with an independent association after adjusting for other cis-eSNPs might be likely to regulate gene expression and found that the possibility of the presence of a gene with an independent cis-eSNP is positively correlated with the number of *cis*-eSNPs in the gene. Based on these studies, a gene with a greater number of eSNPs was assigned as the parent of other genes with fewer eSNPs.
(3) If two genes had the same number of eSNPs and were bi-directional, a directed edge with a higher weight value was selected.
(4) If two genes did not have eSNPs and were bi-directional, a directed edge with a higher weight value was selected.

After constructing four GRNs (i.e., AD GRN, CN_AD_ GRN, CVD GRN, and CN_CVD_ GRN), we calculated the number of child nodes for each parent node in the three GRNs. Next, we calculated the difference between the number of child nodes in the disease (AD or CVD) and the CN GRNs for each parent node. By repeatedly taking random samples of 20% of these results 1000 times, a null distribution for the number of altered edges between the disease and CN GRNs was curated. A parent gene was defined using the number of changed edges in the disease network with a |*z* score| ≥ 1.96, as the significant dysregulated gene.

## 3. Results

### 3.1. Blood Datasets and High Quality Dataset Selection

For the six AD blood datasets, the FC between the AD and AD-matched controls (CN_AD_) for all genes was measured using limma [19]. Spearman’s correlation was used to compare each pair of these six lists of FC values, and the results showed that only a single pair, GSE63060 and GSE63061, exhibited Spearman correlation coefficients (SCC) of more than 0.3 (Figure S1).

Furthermore, for the 11 CVD blood datasets, the FC was calculated for all genes between the CVD and CVD-matched control (CN_CVD_) using limma and then the FC values were compared among all possible pairs in the CVD blood datasets. The following five pairs had an SCC of more than 0.3: GSE60993-GSE20681, GSE60993-GSE59867, GSE60993-GSE66360, GSE20680-GSE20681, and GSE59867-GSE62646 (Figure S1).

**Figure 2:**
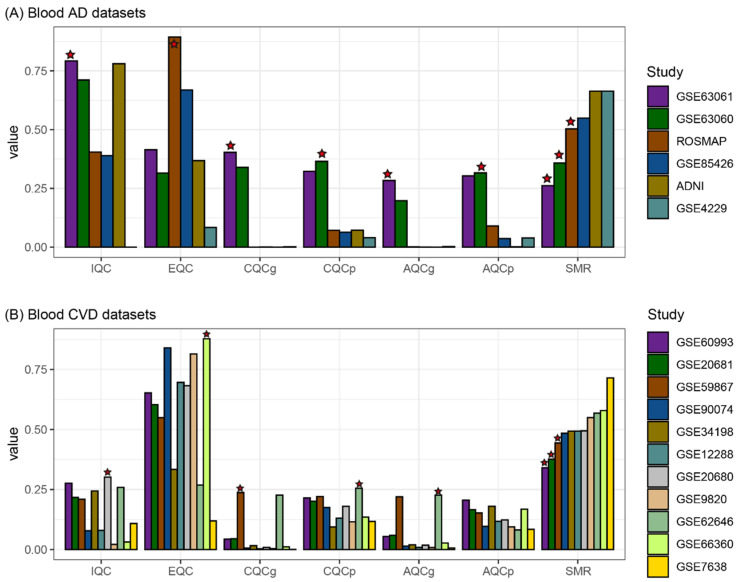
Measurement and comparison of the quality of the blood gene expression datasets using MetaQC. The quality indices were measured by iteratively selecting the four datasets from the six AD blood (**A**) and eleven CVD blood (**B**) gene expression datasets. The heights (y-axis) of each bar plot represents the mean values of each quality index. For example, the quality indices of GSE60993 were measured 120 times (_10_C_3_). The mean values of the 120 measures for each quality index were calculated. Stars denote the first ranking dataset, which means the dataset had the best quality for a specific quality index. AD, Alzheimer’s disease; CVD, cardiovascular disease; IQC, internal quality control; EQC, external quality control; CQCg, consistency quality control (gene); CQCp, consistency quality control (pathway); AQCg, accuracy quality control (gene); AQCp, accuracy quality control (pathway); SMR, standardized mean rank.

The quality indices were measured by iteratively selecting four datasets from the six AD blood gene expression datasets, resulting in 15 cases (_6_C_4_) of running MetaQC. In other words, the dataset had 10 results for each quality index (_5_C_3_). Note that batch normalization was performed separately in each loop of the running MetaQC algorithm. According to the mean value for the SMR, GSE63061, GSE63060, and ROSMAP were ranked as the top three for high quality among the six blood gene expression datasets (Figure 2A). Therefore, GSE63061, GSE63060, and ROSMAP were selected for the construction of a large AD blood dataset from which to select blood AD-related genes. Similarly, from the 11 CVD blood datasets, four datasets were iteratively selected, yielding a CVD dataset with 120 values for each quality index. Based on the SMR values, the GSE60993, GSE20681, and GSE59867 datasets were selected to identify the blood CVD-related genes (Figure 2B).

### 3.2. Identification of the Blood AD-Related Genes

**Figure 3:**
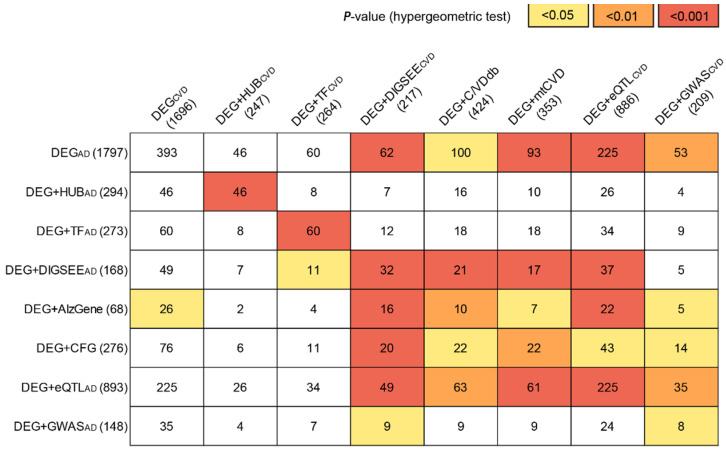
The blood AD- and CVD-related genes. The numbers in parentheses indicate the number of selected candidate DRGs. Numbers in the matrix are the number of common genes between each of the eight-sets of the blood AD- (row) and CVD-related genes (column). DRGs, disease-related genes; AD, Alzheimer’s disease; CVD, cardiovascular disease; DEG, differentially expressed gene; TF, transcription factor; CFG, convergent functional genomics; eQTL, expression quantitative trait loci; GWAS, genome-wide association study; mtCVD, multi-tissue CVD-related genes.

The GSE63061, GSE63060, and ROSMAP datasets comprised 14,477, 14,407, and 15,796 genes (based on the Entrez gene), respectively. A total of 9973 genes were common among the three AD blood datasets. We removed the batch effect among the three AD datasets and integrated them into a large blood AD dataset. A total of 1797 DEGs with an FDR-adjusted *p*-value < 0.05 between the AD and CN_AD_ (DEG_AD_) were identified in the large blood AD dataset (Figure 3).

The 1797 DEGs were mapped onto the PPI network [29] and 278 genes with more than 10 edges were selected as DEG + HUB_AD_ (Figure 3). By conducting an intersection between DEG_AD_ and the 2765 TF-related genes [34], 273 genes were identified as DEG + TF_AD_ (Figure 3). From the DigSee database, a list of 1591 AD-related genes (DIGSEE_AD_) was obtained, and 168 genes were DEG_AD_ and DIGSEE_AD_, which were defined as DEG + DIGSEE_AD_ (Figure 3). A list of 614 AD-related genes were extracted from the AlzGene database [36], of which 68 genes were DEGs, referred to as DEG + AlzGene (Figure 3). We manually curated CFG scores for DEG_AD_ and selected 276 genes with a CFG score ≥ 3 as DEG + CFG (Figure 3). Based on an FDR-adjusted *p*-value < 0.05 for the cis-association between gene expression and SNP, 5647 genes (annotated by Entrez ID) had one or more of the eSNPs. Among the 5647 genes with the blood cis-eQTL evidence, 893 genes were DEG_AD_ and were assigned as DEG + eQTL_AD_ (Figure 3). According to the uncorrected *p*-values < 0.001 for the association between the genetic variants and the presence of AD [39], 15,422 SNPs and their corresponding genes (*n* = 2242) were selected. Among the 2242 genes with GWAS evidence, 148 were DEG_AD_ and were arranged as DEG + GWAS_AD_ (Figure 3).

### 3.3. Identification of the Blood CVD-Related Genes

The selected CVD blood datasets included 137 cases of acute coronary syndrome (ACS), 145 CAD without ACS, and 106 CN_CVD_ samples. After removing the batch effect among the three CVD datasets (GSE60993, GSE20681, and GSE59867), 1696 DEGs were identified based on the three statuses (DEG_CVD_) using the limma package [19]. In line with the process of selecting the blood AD-related genes, 247, 264, 217, and 886 genes were selected as DEG + HUB_CVD_, DEG + TF_CVD_, DEG + DIGSEE_CVD_, and DEG + eQTL_CVD_, respectively (Figure 3).

From C/VDdb [41], a list of 3495 CVD-related genes was extracted, of which 424 were DEG_CVD_ and arranged as DEG + C/VDdb (Figure 3). Previously, from seven tissues in STAGE [42], 171 modules were constructed, of which 28 were further selected based on the CAD heritability in STARNET [44]. A total of 2943 genes were obtained from the 28 modules (mtCVD), of which 353 were DEG_CVD_ and were arranged as DEG + mtCVD (Figure 3). Among the 3245 unique genes that presented GWAS evidence (uncorrected *p*-value < 0.001) [45], 209 genes were DEG_CVD_, which were assigned as DEG + GWAS_CVD_ (Figure 3).

Figure 3 summarizes all sets of AD- and CVD-related genes and the degree of overlap for all possible pairs measured by the hypergeometric test (Supplementary Materials). Among the 64 pairs (8 (AD sets) × 8 (CVD sets)), 30 pairs were significantly overlapped based on a hypergeometric test.

Based on the SCC, we compared the FC values between AD and CN for each of the eight sets of the AD-related genes in the large blood AD dataset with those in the six brain AD gene expression datasets (Supplementary Materials). As a result, the large blood AD dataset was highly positively correlated with the three brain datasets (GSE132903, GSE33000, and GSE5281), and their correlation varied according to the different gene sets (Figure S2A). In the comparison of the large blood AD datasets with 11 blood CVD datasets, six datasets (GSE60993, GSE20681, GSE59867, GSE9820, GSE62646, and GSE66360) had highly positive correlation coefficients, and three datasets were negatively correlated (Figure S2B).

When comparing the FC values for each of the eight sets of the CVD-related genes between the ACS and CN in the large blood CVD dataset with those in the five tissue (heart, vessel, and fat) CVD datasets, two tissue datasets (GSE1869 and GSE43292) showed a positive correlation with significant results based on a permuted *p*-value < 0.05 (Figure S3A). In the comparison between the large blood CVD dataset and the six blood AD datasets, three datasets had positive correlations, of which two (GSE63060 and GSE63061) had significant results for the eight sets of the CVD-related genes (Figure S3B).

### 3.4. Blood AD-Related Genes for Brain AD and Blood CVD Prediction

By consolidating the statistically significant results from the large blood expression datasets and the previously validated biological findings (Figure 1), we identified eight sets (DEG_AD_, DEG + HUB_AD_, DEG + TF_AD_, DEG + DIGSEE_AD_, DEG + AlzGene, DEG + CFG, DEG + eQTL_AD_, and DEG + GWAS_AD_) of blood AD-related genes.

**Figure 4:**
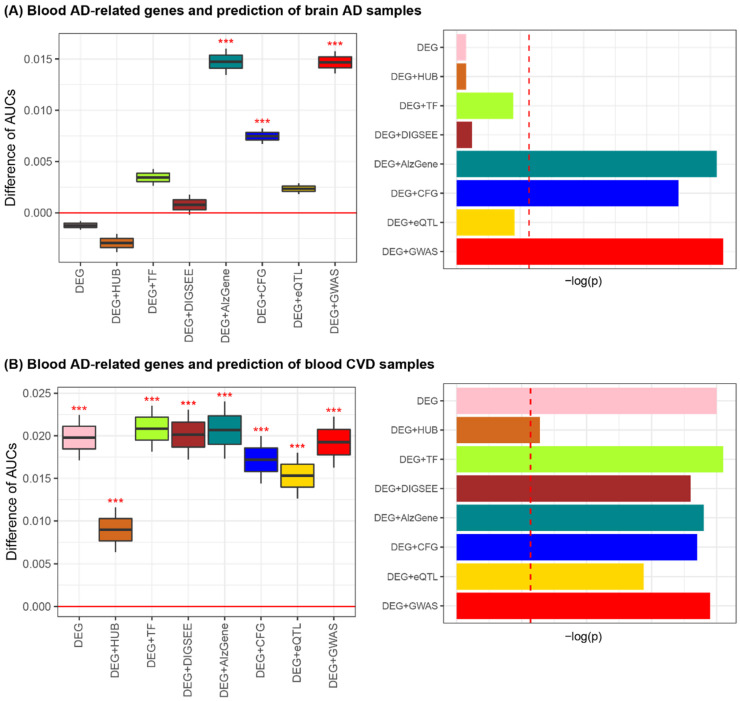
Blood AD-related genes and prediction of the brain AD (**A**) and blood CVD samples (**B**). *** *p* < 0.001. Red dashed vertical lines in the bar plots on the right-side indicate the value of −log(Bonferroni-corrected *p*-value of 0.05 = nominal *p*-value of 0.00625 (=0.05/8)). AD, Alzheimer’s disease; CVD, cardiovascular disease; AUC, area under the receiver operating characteristic curve; DEG, differentially expressed gene; TF, transcription factor; CFG, convergent functional genomics; eQTL, expression quantitative trait loci; GWAS, genome-wide association study.

Then, the actual DRGs were selected by comparing the predictive accuracy of the model established by the candidate DRGs with that by the randomly selected genes. For the eight lists of the blood AD-related genes and matched random sets of genes, the prediction performance was investigated on the six AD brain datasets. Performing 1000 iterations of the brain AD predictions for each of the six datasets, we obtained 6000 AUCs for each of the eight lists of the blood AD-related genes and 6000 AUCs from the matched-random gene sets. Of the eight cases of AD-related genes, three cases (DEG + AlzGene, DEG + CFG, and DEG + GWAS_AD_) exhibited improved performance for AD classification compared to the matched random cases (Figure 4A and Figure S4).

The prediction performance on the 11 blood CVD blood datasets was evaluated using the eight lists of blood AD-related genes. With 1000 iterations of blood CVD predictions for each of the 11 datasets, we obtained 11,000 AUCs for each of the eight blood AD-related gene sets. As a result, all cases exhibited better performance in discriminating blood CVD samples than those generated by the matched random gene sets (Figure 4B and Figure S5). Collectively, DEG + AlzGene, DEG + CFG, and DEG + GWAS_AD_ were highly informative for both brain AD and blood CVD prediction.

### 3.5. Blood CVD-Related Genes for Tissue CVD and Blood AD Prediction

**Figure 5:**
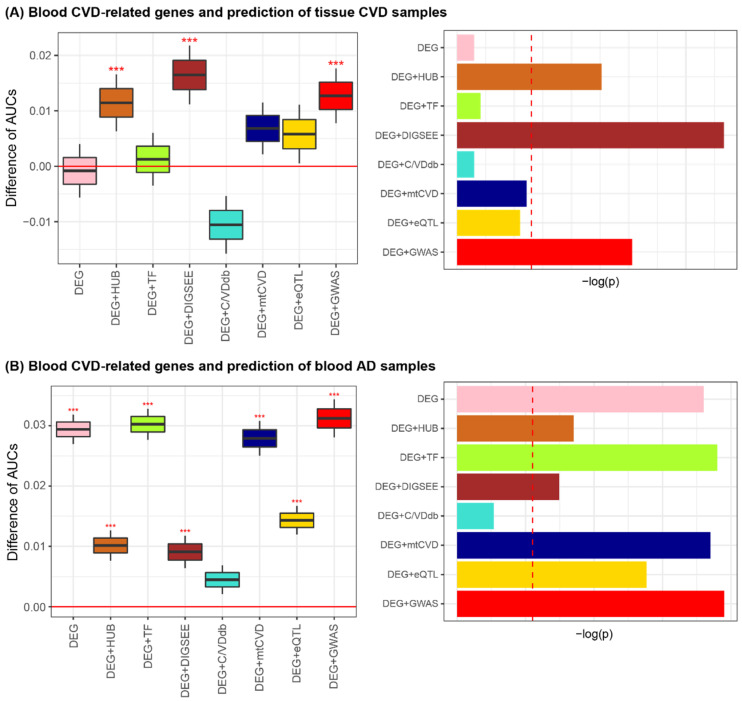
Blood CVD-related genes and predictions for tissue CVD (**A**) and blood AD samples (**B**). *** *p* < 0.001. Red dashed vertical lines in the bar plots on the right-side indicate the value of −log(Bonferroni-corrected *p*-value of 0.05 = nominal *p*-value of 0.00625 (=0.05/8)). AD, Alzheimer’s disease; CVD, cardiovascular disease; AUC, area under the receiver operating characteristic curve; DEG, differentially expressed gene; TF, transcription factor; mtCVD, multi-tissue CVD-related genes; eQLT, expression quantitative trait loci; GWAS, genome-wide association study.

Similar to the identification of the eight sets of blood AD-related genes, the eight lists of blood CVD-related genes were curated by considering statistical methods and domain knowledge (e.g., PPI network, TF database, and disease- and expression-related SNPs). For the eight CVD-related gene sets, we investigated the prediction performance of three types of CVD tissue (heart, fat, and vessel) samples. Using 1000 iterations of the tissue CVD predictions for each of the five datasets, 5000 AUCs were obtained for each of the eight lists of the blood CVD-related genes and 5000 AUCs were obtained from a matched random gene set. As a result, of the eight blood CVD-related gene sets, three (DEG + HUB_CVD_, DEG + DIGSEE_CVD_, and DEG + GWAS_CVD_) exhibited improved performance for tissue CVD classification when compared to the matched random cases (Figure 5A and Figure S6).

The prediction performance on the six blood AD datasets was evaluated using the eight lists for the blood CVD-related genes as input features. With 1000 iterations of these prediction tasks for the six datasets, 6000 AUCs were obtained for each of the eight CVD-related gene sets. Of the eight cases of the CVD-related genes, all except for DEG + C/VDdb showed improved performance for the blood AD classification compared to those obtained by the matched random cases. Collectively, we determined that DEG + HUB_CVD_, DEG + DIGSEE_CVD_, and DEG + GWAS_CVD_ were informative blood CVD-related genes for both tissue CVD and blood AD prediction (Figure 5B and Figure S7).

### 3.6. Comparison of DRGs Obtained from the Blood Microarrays and Tissue (Brain or Heart) Single Cell RNA-Sequencing Datasets

**Figure 6:**
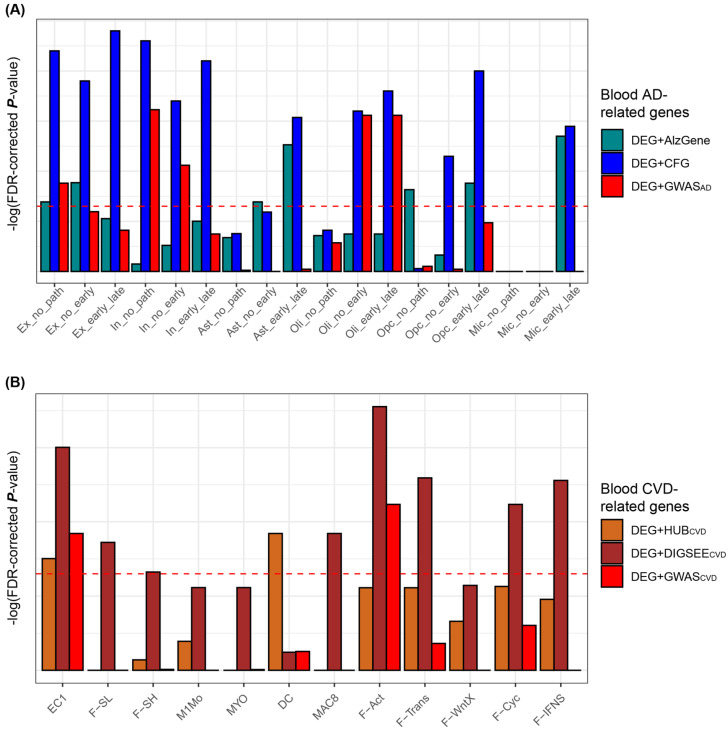
Comparison of the blood and the single-cell-based tissue DRGs. Red horizontal lines indicate an FDR-corrected *p*-value < 0.05, as measured by Fisher’s exact test. (**A**) “no_path”, “no_early”, and “early_late” indicate no pathology vs. AD pathology (early and late AD), no pathology AD vs. early AD, and early vs. late AD, respectively. (**B**) Twelve groups after excluding 23 insignificant cases out of 35 clusters of cell type-specific DEGs obtained from CVD mouse hearts are used for the comparisons. Horizontal lines in A and B indicate an FDR-adjusted *p*-values < 0.05, as measured using Fisher’s exact test. AD, Alzheimer’s disease; CVD, cardiovascular disease; AUC, area under the receiver operating characteristic curve; DEG, differentially expressed gene; TF, transcription factor; CFG, convergent functional genomics; eQLT, expression quantitative trait loci; GWAS, genome-wide association study; Ex, excitatory neurons; In, inhibitory neuron; Ast, astrocyte; Oli, oligodendrocyte; Opc, oligodendrocyte progenitor cell; Mic, microglia; EC, Endothelial cel; F-SL, fibroblast-*Sca1*-low; F-SH, fibroblast-*Sca1*-high; M1Mo, M1 monocyte; MYO, Myofibroblast; DC, dendritic cell; MAC, macrophage; F-Act, fibroblast-activated; F-trans, fibroblast-transitory; F-WntX, fibroblast-Wnt expressing; F-Cyc, fibroblast-cycling; F-IFNS, fibroblast-IFN stimulated.

The blood DRGs obtained from the blood bulk transcriptomic datasets were compared with the previously validated tissue (i.e., brain and heart) DRGs obtained from the brain and heart single cell analyses. From the brain AD single cell analyses [46], we curated 18 lists of DEGs consisting of six cell types and three types of comparisons with an FDR-adjusted *p*-value < 0.05 (Figure 6A). Three preliminarily selected lists of blood AD-related genes were compared with the 18 lists for the single cell-based AD-related genes. According to Fisher’s exact test, the DEG + CFG of the three blood AD-related gene sets showed significant enrichment in 12 of the 18 lists of the cell type-specific DRGs (Figure 6A).

For the heart CVD single-cell analyses, the gene expression dataset by Farbehi et al. [47] was analyzed. Single-cell analyses were performed twice (TIP and GFP), from which we curated 24 and 11 lists for the single-cell-based DEGs between the two conditions, respectively. Based on Fisher’s exact test, the eight lists of the blood CVD-related genes were compared with the 35 lists of the cell type-specific DEGs, of which we removed the 23 lists that had insignificant associations with most of the eight blood CVD-related gene sets to avoid complicating the visualization of the results more than necessary (data not shown). As a result, 12 lists of the single-cell-based DRGs were generated and compared with three preliminarily selected lists of the blood CVD-related genes, resulting in the DEG + DIGSEE_CVD_ significantly overlapping with eight of the 12 lists of single-cell-based DRGs (Figure 6B).

DEG + CFG was selected as the AD-related genes that accurately predicted the performance of the brain AD and the blood CVD samples and exhibited significant enrichment with the cell type-specific DEGs between AD and CN (Table S1). Similarly, DEG + DIGSEE_CVD_ was selected as the actual DRGs due to the improved performance of tissue CVD and blood AD prediction and the significant enrichment with the heart CVD-related genes from the single-cell-based analyses (Table S2).

**Figure 7:**
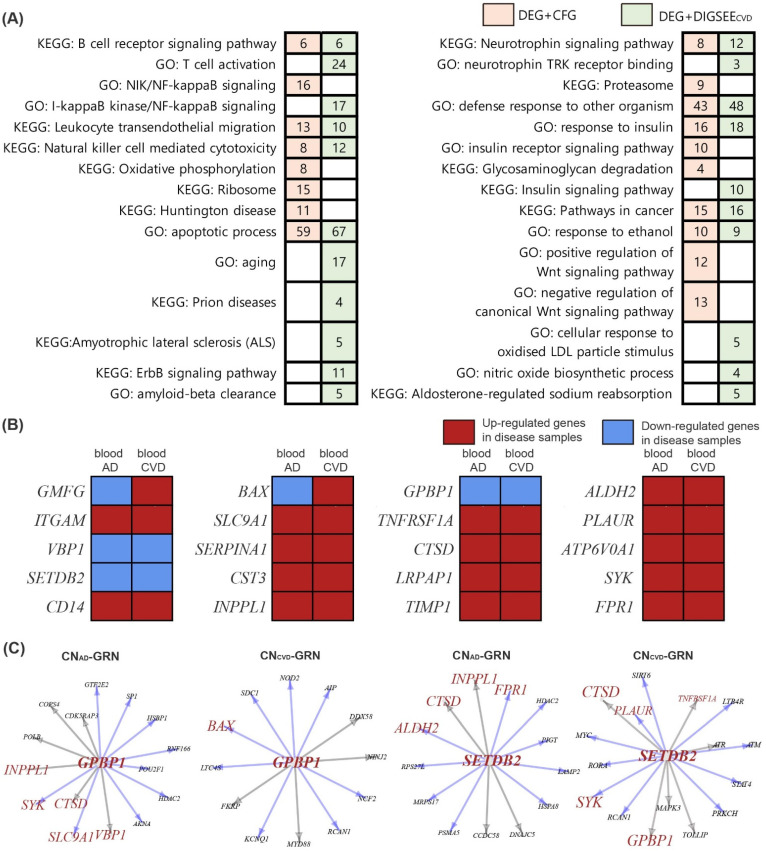
Biological functions and differential and regulatory patterns of the blood AD- (DEG + CFG) and CVD-related genes (DEG + DIGSEE_CVD_). (**A**) Enriched pathways in DEG + CFG and DEG + DIGSEE_CVD_ genes. Numbers in the colored rectangles indicate the number of the common genes between the DRGs and genes in each biological pathway. (**B**) Common genes between the DEG + CFG and DEG + DIGSEE_CVD_ genes. The blood AD and CVD datasets are the integrated datasets obtained from three blood AD (GSE63061, GSE63060, and ROSMAP) and three blood CVD datasets (GSE60993, GSE20681, and GSE59867), respectively. (**C**) Transcription factors *GPBP1* and *SETDB2* have decreased edges with child genes in both AD and CVD networks. Brown-colored genes indicate the common genes between DEG + CFG and DEG + DIGSEE_CVD_. Blue arrows indicate the edges that disappear in the disease GRN. Grey arrows denote the edges that are simultaneously present in the disease and CN GRNs. AD, Alzheimer’s disease; CVD, cardiovascular disease; CN, control; DEG, differentially expressed gene; CFG, convergent functional genomics; GO, gene ontology; KEGG, Kyoto Encyclopedia of Genes and Genomes; GRN, gene regulatory network.

The 259 pathways enriched by genes in the DEG + CFG included the AD-related pathways, such as the B cell receptor signaling pathway, NIK/NF-kappaB signaling, oxidative phosphorylation, ribosome, proteasome, and positive or negative regulation of Wnt signaling pathway (Figure 7A). DEG + DIGSEE_CVD_ involved 503 biological pathways, consisting of the following CVD-related pathways: T cell activation, ErbB signaling pathway, amyloid-beta clearance, cellular response to oxidized LDL particle stimulus, and aldosterone-regulated sodium reabsorption (Figure 7A). We compared pathways enriched by DEG + CFG and DEG + DIGSEE_CVD_; 60 pathways were shared (*p*-value by hypergeometric test: 2.14 × 10 ^−25^). When comparing DEG + CFG and DEG + DIGSEE_CVD_, 20 genes (*GMFG*, *ITGAM*, *VBP1*, *SETDB2*, *CD14*, *BAX*, *SLC9A1*, *SERPINA1*, *CST3*, *INPPL1*, *GPBP1*, *TNFRSF1A*, *CTSD*, *LRPAP1*, *TIMP1*, *ALDH2*, *PLAUR*, *ATP6V0A1*, *SYK*, *FPR1*) were common (*p*-value by hypergeometric test: 5.06 × 10^−5^, Figure 3 and Figure 7B).

### 3.7. Gene Regulatory Network and Identification of Altered Genes in the Disease Network

We constructed AD, CVD, and matched CN GRNs separately and compared them to identify disease-related regulatory patterns between the genes. Blood AD and CN samples from GSE63061, GSE63060, and ROSMAP were used to establish the AD and CN_AD_ GRNs, respectively. Blood CVD samples in GSE60993, GSE20681, and GSE59867 were used to construct the CVD GRN. Note that blood CN_CVD_ samples from the two datasets except for GSE59867 were used to establish the CN_CVD_ GRN. Therefore, the DRGs in DEG + CFG (*n* = 276) and DEG + DIGSEE_CVD_ (*n* = 217) were used as the background genes for the AD and CVD networks establishment, respectively. Among the DRGs in DEG + CFG and DEG + DIGSEE_CVD_, 39 and 45 were the known TFs [34], respectively. Using GENIE3, we constructed AD and CVD GRNs with setting the 39 and 45 TFs as prior, respectively, and removed false-positive results by using blood cis-eQTL evidence as described in the Methods section [38], yielding AD GRN (728 edges and 319 genes), CN_AD_ GRN (720 edges and 318 genes), CVD GRN (460 edges and 242 genes), and CN_AD_ GRN (583 edges and 246 genes).

The AD and CVD GRNs included 39 and 45 parent genes with one or more child genes, respectively. With a cut-off (|*z* score| ≥ 1.96) for the sampling distribution of the altered number interactions between the networks, 20 genes were selected among the 39 parent genes that had a significantly changed number of child genes in the AD GRN compared to those in the CN GRN (Table S3). In case of the CVD GRN, 25 parent genes among the 45 parent genes were significantly dysregulated compared to the CN GRN (Table S3). Comparing the 20 dysregulated TFs in the AD GRN and the 25 altered TFs in the CVD GRN, two genes (*GPBP1* and *SETDB2*) overlapped, which had decreased edges with child genes in both the AD and CVD networks, compared to the control network (Figure 7C).

For the two dysregulated upstream genes, we analyzed how these two common regulators between AD and CVD GRNs differentially regulated the 20 common genes between the DEG + CFG and DEG + DIGSEE_CVD_ in the disease network, compared to the CN network (Figure 7C). *GPBP1* lost its regulatory interaction with two genes (*SYK* and *SLC9A1*) and one gene (*BAX*) among the 20 common genes in the AD and CVD GRN, respectively, compared to the matched CN GRNs (Figure 7C). In addition, *SETDB2* exhibited the disappearance of edges with two genes (*ALDH2* and *FPR1*) and two genes (*SYK* and *PLAUR*) in the AD and CVD GRN (Figure 7C), respectively.

## 4. Discussion

This study identified the blood AD- and CVD-related genes using a statistical method (i.e., selection of genes with significantly different expression levels between two conditions), a PPI network, TF database, disease–gene relationship databases, as well as the validated SNPs related to expression or disease status. Among the candidate sets for the DRGs, we selected the blood DRGs with improved prediction performance for the other tissue and the same disease and the same tissue and other diseases, when compared to the matched, random sampling genes. Moreover, we selected the blood DRGs that significantly overlapped with most cell type-specific DRGs obtained from the brain and heart single cells, finally resulting in DEG + CFG and DEG + DIGSEE_CVD_ as the actual blood AD- and CVD-related genes, respectively. Using the AD-CFG database, Xu et al. [37] revealed that the *YAP1* gene is a crucial regulator of AD. Furthermore, previous work found that the blood AD-related genes obtained from the AD-CFG database exhibited high performance for blood AD prediction among the different cohorts [30]. Using DIGSEE [35], Park et al. [52] identified several genes that have somatic mutations directly associated with the phosphorylation of the tau protein. Although DIGSEE has been used to identify the pathophysiology of neurodegenerative diseases [52,53], limited studies have used this search engine to elucidate the mechanisms of cerebro-cardiovascular disease.

In the disease (AD and CVD) network, we identified two upstream genes with the disappearance of interaction with other genes, compared to the CN network (*GPBP1* and *SETDB2*). *GPBP1*, also called *Vasculin*, is reported to be expressed in the vascular wall and plasma and its expression in plasma plays a crucial role in atherosclerosis [54]. Ong et al. [55] found that the gene expression of *GPBP1* is significantly down-regulated in cerebral artery of rabbits exposed to hypertension and/or hypercholesterolemia. In a study analyzing AD brain single cells [46], *GPBP1* was dysregulated in AD patient’s inhibitory and excitatory neurons.

*SETDB2*, a *KMT1* subfamily of *SET*-domain-containing lysine methyltransferases, is known to play a crucial role in lipid metabolism via the glucocorticoid-dependent pathway [56]. Moreover, *SETDB2* is linked to an anti-inflammatory response via regulation of lipopolysaccharide and interferon-induced genes [57,58]. Similarly, a study suggested that *SETDB2* was related to neuroinflammation, which is a risk factor for AD dementia [59]. *SETDB2* was also reported to be associated with the atherosclerotic change in a monkey’s iliac artery [60]. Two common putative TFs (*GPBP1* and *SETDB2*) and their association with AD and CVD have been reported as candidates from the results of the putative or computational analysis, a finding not validated by pinpointed analyses that observe the downstream mechanism affected by the dysregulation of upstream genes (i.e., *GPBP1* and *SETDB2*). Moreover, these lines of evidence had been derived from the association of these genes with one disease (i.e., AD or CVD), but not from those with co-occurrence of AD and CVD. Collectively, the shared downstream pathways affected by the dysregulation of *GPBP1* and *SETDB2* between AD and CVD remain unknown. Future studies that analyze subjects with co-occurrence of AD and CVD are warranted to identify the common or differential pathogenesis triggered by the altered function of *GPBP1* and *SETDB2* between AD and CVD.

Among the 20 common genes between DEG + CFG and DEG + DIGSEE_CVD_, SYK had a decreased interaction with two upstream genes (GPBP1 and SETDB2) in the disease GRNs (Figure 7). SYK plays a crucial role in adaptive immunity, innate immune recognition, platelet activation, cellular adhesion, and vascular development [61]. Recently, SYK has been reported to mediate microglial activation and neurotoxicity by regulating NF-κB and glycogen synthase 3β [62]. In addition, several SYK inhibitors have been proposed as potential treatments for AD as well as MI [63,64].

Several mechanisms are associated with AD, of which the amyloid beta cascade, including the cleavage of amyloid precursor protein (APP), the formation of toxic oligomers, and the development of beta sheet and plaque, are considered the core AD pathology. As potential therapeutic strategies for the amyloid beta cascade, several compounds (e.g., Bapineuzumab, glucagon-like peptide, statins, ibuprofen, and naturally obtained dietary flavonoids) have been introduced [65]. The gene expressions of *GPBP1* and *SETDB2* were reported to be correlated with the levels of *tau* protein in AD mice [37]. Moreover, other pathophysiological mechanisms, such as mitochondrial dysfunction, excitotoxicity, oxidative stress, and neuroinflammation, are also associated with the onset of AD, hence several alternative treatments targeting these pathways have been developed [66]. We identified *SYK*, an inflammation-related gene [62], as the actual DRG. As the common pathological mechanism between AD and CVD, a cholesterol mechanism has been proposed [67,68]. Recently, the superfamily of ATP-binding cassette (*ABC*) transporters has been reported to have an interplay in amyloid beta translocation and cholesterol metabolism [69]. Similarly, accumulating evidence suggests that *GPBP1* may be involved in cholesterol metabolism [54,55]. Among the diagnostic and therapeutic applications, our study contributes to the early and accurate detection of patients with high AD or CVD risk since our overall findings (e.g., AD- or CVD-related genes) were obtained from blood tissues and their combinatory effect was validated via the classification performance.

## 5. Conclusions

By considering the statistical results, the PPI network, TF database, disease–gene relationship database, and the eQTL and GWAS evidence, several sets of DRGs were identified. Moreover, by performing classification tasks and comparative analyses with disease-related signatures of the brain and heart single-cell RNA sequencing, DEG + CFG and DEG + DIGSEE_CVD_ were selected as the actual DRGs. Finally, two commonly dysregulated upstream genes between AD and CVD were identified by establishing GRNs, which provides further insights into the shared pathophysiology between neurodegenerative and atherosclerotic diseases.

## Data Availability

Gene expression datasets are publicly available (ADNI, http://adni.loni.usc.edu/; GEO, https://www.ncbi.nlm.nih.gov/geo/; ArrayExpress, https://www.ebi.ac.uk/arrayexpress/ (accessed on 15 December 2020)). A complete listing of ADNI investigators can be found at: http://adni.loni.usc.edu/wp-content/uploads/how_to_apply/ADNI_Acknowledgement_List.pdf.

## Supplementary Materials

The following are available online at https://www.mdpi.com/article/10.3390/biomedicines9111525/s1. Figure S1: Comparison of disease-related signatures among the blood gene expression datasets. Figure S2: Comparison of disease-related transcriptomic signatures for the selected AD-related genes obtained from the large blood AD dataset with those of the brain AD and blood CVD datasets. Figure S3: Comparison of disease-related transcriptomic signatures for the selected CVD-related genes obtained from the large blood CVD dataset with those of the tissue CVD and blood AD datasets. Figure S4: Performance of the eight blood AD-related gene sets for the prediction of brain AD. Figure S5: Performance of the eight blood AD-related gene sets for the prediction of blood CVD. Figure S6: Performance of the eight blood CVD-related gene sets for the prediction of CVD tissues (heart, vessel, and fat) CVD. Figure S7: Performance of the eight blood CVD-related gene sets for the prediction of blood AD. Supplementary Table S1: Genes in DEG + CFG. Supplementary Table S2: Genes in DEG + DIGSEE_CVD_. Supplementary Table S3: Parent genes with a significantly changed number of child genes in disease gene regulatory network.
