# Supplementary material for: Identification of Disease-Related Genes That Are Common between Alzheimer’s and Cardiovascular Disease Using Blood Genome-Wide Transcriptome Analysis
Source: Biomedicines. 2021 Oct 23;9(11):1525. doi: 10.3390/biomedicines9111525 (PMC8614900; doi:10.3390/biomedicines9111525)
Supplement: Supplementary file 1 [file biomedicines-09-01525-s001.zip › biomedicines-1404235-supplementary.pdf]

Identification of disease-related genes that are common between Alzheimer's and cardiovascular disease using blood genome-wide transcriptome analysis

(Supplementary Material)

Taesic Lee[1,2], Hyunju Lee[1,3,*]

[1] Department of Biomedical Science and Engineering, Gwangju Institute of Science and Technology, Gwangju, South Korea.

[2] Department of Family Medicine, Yonsei University Wonju College of Medicine, Wonju, South Korea

[3] School of Electrical Engineering and Computer Science, Gwangju Institute of Science and Technology, Gwangju, South Korea.

***Corresponding author**

E-mail: hyunjulee@gist.ac.kr

Blood AD Datasets (For Section 2.1 in the main text)

AddNeuroMed

We downloaded the preprocessed (normalization) AddNeuroMed datasets (Accession ID: GSE63060 and GSE63061) from the Gene Expression Omnibus database (GEO) [1]. GSE63060 included 145 AD and 104 CN blood samples, whereas GSE63061 comprised 139 AD and 134 CN blood samples. Microarray expression analyses of GSE63060 and GSE63061 were conducted using the Illumina HumanHT-12 V3.0 expression beadchip and Illumina HumanHT-12 V4.0 expression beadchip, respectively. RNA expression values in the datasets (GSE63060 and GSE63061) underwent variance-stabilizing transformation, followed by quantile normalization. Following characteristics were included in these datasets: ethnicity, age, and gender.

ROSMAP

We downloaded the ROSMAP dataset from the AD Knowledge Portal [2-4]. We performed read alignment via STAR [5], and the gene expression was quantified using RSEM [6]. We scaled the FPKM values via log-transformation and subjected them to quantile normalization. Furthermore, we evaluated the degree of mapping between input reads and the RefSeq hg19 reference genome. We removed two samples with less than 0.6 of the ratio of the number of mapping reads to that of the input reads, yielding 122 AD and 282 CN blood samples. Gene expression for all samples were sequenced via a HiSeq 2500 (Illumina) or a NovaSeq 6000 (Illumina). Thereby, we removed the batch effect between two sequencing methods using ComBat algorithm [7].

GSE85426

We downloaded "GSE85426 raw data.txt" from the GEO [1]. The GSE85426 dataset comprised 90 AD and 90 CN blood samples. Gene expression was measure using the platform of Agilent-028004 SurePrint G3 Human GE 8x60K Microarray. We scaled and normalized the gene expression values in GSE85426 via exponential function and quantile normalization, respectively. Following characteristics were included in this dataset: age and gender.

ADNI

We downloaded the preprocessed (normalization) ADNI expression data normalized by the robust multi-array analysis (RMA) method [8]. Disease status of samples in ADNI differed according to the baseline and the date of blood examination for RNA expression. Therefore, we curated the disease status (AD or CN) from diagnosis at the time that was same or closest with those at the blood RNA examination. We excluded samples with RNA integrity number (RIN) less than 6.9 as other study used [9]. As a result, we obtained 63 AD and 136 blood samples. RNA expression values in the ADNI underwent Robust Multichip Average (RMA) [8].

GSE4229

We downloaded the GSE4229 dataset that included 22 AD and 18 CN blood samples. Microarray expression analyses of GSE4229 were conducted using the NIA MGC, Mammalian Genome Collection. Thereafter, we scaled and normalized RNA expression values in GSE4229 via log-transformation and quantile normalization, respectively.

Brain AD Datasets (For Section 2.1 in the main text)

GSE118553

We downloaded the preprocessed (normalization) GSE118553 from the Gene Expression Omnibus database (GEO) [1]. GSE118553 included 301 AD and 100 CN brain samples. Microarray expression analyses were conducted using the Illumina HumanHT-12 V4.0 expression beadchip. RNA expression values in the GSE118553 underwent Maximum Likelihood Estimation (MLE) background corrected, log2 transformed, and Robust Spline Normalization (RSN). Following characteristics were included in this dataset: age and gender.

GSE132903

We downloaded the preprocessed (normalization) GSE132903 dataset that included 97 AD and 98 CN brain samples. Microarray expression analyses were conducted using the Illumina HumanHT-12 V4.0 expression beadchip. RNA expression values in the GSE132903 underwent variance-stabilizing transformation, followed by quantile normalization. Following characteristics were included in this dataset: age and gender.

GSE33000

We downloaded GSE33000 dataset that included 310 AD and 157 CN brain samples. Microarray expression analyses were conducted using the Rosetta/Merck Human 44k 1.1 microarray. We scaled and normalized the gene expression values in GSE33000 via exponential function and quantile normalization, respectively. Following characteristics were included in this dataset: age and gender.

GSE5281

We downloaded GSE5281 dataset that included 87 AD and 74 CN brain samples. Microarray expression analyses were conducted using the Affymetrix Human Genome U133 Plus 2.0 Array. We scaled and normalized the gene expression values in GSE5281 via log-transformation and quantile normalization, respectively. Following characteristics were included in this dataset: age, gender, cell type, ethnicity, developmental stage, and genetic variation.

GSE84422

We downloaded the preprocessed (normalization) GSE84422 dataset that included 1474 AD and 428 CN brain samples. Microarray expression analyses were conducted using the Affymetrix Human Genome U133A Array (GPL96) or Affymetrix Human Genome U133B Array (GPL97). RNA expression values in the GSE84422 underwent RMA [8]. Following characteristics were included in this dataset: age, gender, ethnicity (race), postmortem interval minutes, pH, clinical dementia rating (CDR), Braak neurofibrillary tangle score, neuropathological category, average neuritic plaque density, sum of CERAD rating scores in multiple brain regions, and sum of neurofibrillary tangles density in multiple brain regions.

Blood CVD Datasets (For Section 2.1 in the main text)

GSE60993

We downloaded the preprocessed (normalization) GSE60993 dataset that included 26 acute coronary syndrome (ACS) and 7 CN blood samples. Microarray expression analyses were conducted using the Illumina HumanWG-6 v3.0 expression beadchip. RNA expression values in the GSE60993 underwent quantile normalization.

PREDICT Trial (GSE20681)

This data (GSE20681) was one of sub-series of GSE20686. Blood samples were obtained from Predict cohort, a multi-center US study. This data included 99 control samples with <50% stenosis in all major coronary vessels and 99 case samples with >50% stenosis in at least one major vessel. Furthermore, this data consisted of individual information, such as age, gender, and smoking status. Microarray samples were labeled and hybridized to Agilent-014850 Whole Human Genome Microarray 4x44K G4112F using the manufacturer's protocol. We normalized this dataset via quantile normalization.

GSE59867

We downloaded GSE59867 dataset that included 111 acute coronary syndrome (ACS) and 46 stable coronary artery (CAD) blood samples. Microarray expression analyses were conducted using the Affymetrix Human Gene 1.0 ST Array. We normalized RNA expression values in the GSE59867 via quantile normalization. Following characteristics were included in this dataset: time at sample collection and HF progression.

GSE90074

We downloaded the GSE90074 dataset comprising 76 CVD and 12 CN blood samples. Microarray expression analyses were conducted using the Agilent-014850 Whole Human Genome Microarray 4x44K G4112F. We scaled gene expression value of this dataset via exponential function, followed by plus 1 and log-transformation to change all values to positive levels. Afterwards, we normalized these values via quantile normalization. Following characteristics were included in this dataset: age, gender, ancestry, obstructive CAD, CAD class, *CXCL5* rank, body mass index (BMI), diabetes, hyperlipidemia, hypertension, and batch ID.

GSE34198

We downloaded the GSE34198 dataset comprising 48 CVD and 49 CN blood samples. Microarray expression analyses were conducted using the Illumina human-6 v2.0 expression beadchip. The minimum gene expression value of GSE34198 was –26.54; therefore, we changed gene expression values as positive values by adding 27.6 to all expression values. Next, we scaled and normalized the gene expression values via log-transformation and quantile normalization, respectively. Following characteristics were included in this dataset: age, gender, diabetes, smoking, height, weight, systolic and diastolic BP, ACEI, β-blocker, diuretic, Ca blocker, statin, fibrate, and other medication.

GSE12288

We downloaded the GSE12288 dataset comprising 110 CVD and 112 CN blood samples. Microarray expression analyses were conducted using the Affymetrix Human Genome U133A Array. We scaled and normalized the gene expression values via log-transformation and quantile normalization, respectively. Following characteristics were included in this dataset: age, gender, ethnicity, and CAD index.

Cathgen Registry (GSE20680)

This data (GSE20680) was one of sub-series of GSE20686. Blood samples were obtained from Cathgen Registry. This data included 52 control samples with ≤ 25% stenosis in all major coronary vessels and

87 case samples with ≥ 50% 70% stenosis in >1 major vessel or ≥50% stenosis in >2 arteries. Microarray samples were labeled and hybridized to Agilent-014850 Whole Human Genome Microarray 4x44K G4112F using the manufacturer's protocol. We normalized this dataset via quantile normalization.

GSE9820
We downloaded the preprocessed (normalization) GSE9820 dataset comprising 19 CVD and 15 CN blood samples. Microarray expression analyses were conducted using the Illumina humanRef-8 v2.0 expression beadchip. RNA expression values in the GSE9820 underwent log-transformation, followed by quantile normalization. Following characteristics were included in this dataset: age and gender.

GSE62646
We downloaded the preprocessed (normalization) GSE62646 dataset comprising 28 ACS and 14 stable CAD blood samples. Microarray expression analyses were conducted using the Affymetrix Human Gene 1.0 ST Array. RNA expression values in the GSE62646 underwent RMA.

GSE66360
We downloaded the preprocessed (normalization) GSE66360 dataset comprising 49 ACS and 50 CN blood samples. Microarray expression analyses were conducted using the Affymetrix Human Genome U133 Plus 2.0 Array. RNA expression values in the GSE66360 underwent RMA. Following characteristics were included in this dataset: cell type.

GSE7638
We downloaded the GSE7638 dataset comprising 110 CVD and 50 CN blood samples. Microarray expression analyses were conducted using the Affymetrix Human Genome U133A 2.0 Array. We normalized the gene expression values via quantile normalization. Following characteristics were included in this dataset: age, gender, and collateral flow index.

Tissue CVD Datasets (For Section 2.1 in the main text)

GSE1869 (heart CVD dataset)

Heart samples in the preprocessed (normalization) GSE1869 were obtained from two institutes: 1) Johns Hopkins Hospital in Baltimore and University of Minnesota in Minneapolis [10]. Specifically, we used samples with dilated cardiomyopathy (DCM). There were the two major forms of DCM, including non-ischemic DCM (NICM) and ischemic DCM (ICM). We arranged 21 samples with NICM and 10 samples with ICM as control and disease group, respectively. Gene expression was measure using the platform of Affymetrix Human Genome U133A Array and underwent RMA normalization.

GSE43292 (carotid vessel CVD dataset)

We downloaded the preprocessed (normalization) GSE43292 dataset comprising 32 atheroma plaque and 32 macroscopically intact tissue samples. Microarray expression analyses were conducted using the Affymetrix Human Gene 1.0 ST Array. RNA expression values in the GSE43292 underwent RMA. Following characteristics were included in this dataset: disease state.

GSE5406 (heart CVD dataset)

The preprocessed (normalization) GSE5406 consisted of three status of myocardium tissue: non-failing, idiopathic DCM, and ischemic ICM hearts [11]. Specifically, we used 16 non-failing heart samples and 108 heart samples with ICM as control and disease statuses, respectively. Probes were then hybridized with individual Affymetrix HU133A arrays according to the manufacturer's instructions. Data underwent RMA normalization.

MAGNet consortium (GSE57338, heart CVD dataset)

We downloaded the preprocessed (normalization) GSE57338 from the GEO [1] including 136 samples with non-failing hearts and 95 samples with heart failure due to ischemic heart disease [12]. RNA expression values in the dataset (GSE57338) was previously background-corrected and normalized following methods: RMA method and an invariant set method. Following characteristics were included in this dataset: age, gender, and heart failure.

GSE64554 (fat CVD dataset)

We downloaded the preprocessed (normalization) GSE64554 from the GEO [1] including 20 fat samples without any history and evidences of coronary artery disease (CAD) and carotid atherosclerosis and 26 samples that underwent coronary artery bypass graft surgery [13]. Fat tissue included epicardial adipose tissue (EAT) and subcutaneous adipose tissue. Microarray expression analyses were conducted using the Illumina HumanHT-12 V3.0 expression beadchip. The GSE64554 underwent quantile normalization with Illumina Genome Studio Software. Following characteristics were included in this dataset: age.

Preprocessing (For Section 2.1 in the main text)

For RNA expression datasets comprising more than 40,000 probes (probe-sets, transcripts), we excluded 30% probes presenting small standard deviations across samples, and 30% of the under-expressed probes based on the small mean expression values across subjects. For the gene expression dataset with less than 40,000 and more than 20,000 probes, we removed 20% probes with small variance across samples, and 20% of probes with small mean values. For datasets with less than 20,000 probes, we determined the cut-off value of 10%. To systemically compare the gene expression of different platforms, we mapped all probes or probe-sets ID to Entrez IDs, and eliminated probes without matched Entrez IDs. The RNA-sequencing dataset had transcripts based on ENSEMBL IDs; therefore, we remapped the ENSEMBL IDs to Entrez ID. For multiple probes, probe-sets, or transcripts annotated with a gene (Entrez ID), we selected the probe with maximum average expression value via "collapseRows" function in Weighted Gene Co-Expression Network Analysis (WGCNA) package [14].

Evaluating classification performance of DRGs (For Section 2.6 in the main text)

We previously compared the prediction performances based on the type of classification models and feature selection methods [9]. However, in the present study, we did not focus on which classifiers produced the good predictive performance of diseases, but on which combinations of genes (feature selection method) proposed good classification performance. Therefore, for the classification model, we fixed an support vector machine (SVM) with setting 1 and 1/(dimension of input features) as cost and gamma values, respectively, to compare the disease prediction performances obtained by different sets of disease-related genes (DRGs). We performed the following tasks to evaluate which the candidate sets of DRGs are accurate and informative in the disease classification.

Step 1) We randomly assigned a gene expression dataset with a ratio of 0.7 and 0.3 ratio into training and testing sets, respectively.

Step 2) Using a candidate gene set (one of the candidate sets of DRGs) and SVM as input features and classification model, we constructed a prediction model, and measured the classification performance of AD or CVD patients in the testing set.

Step 3) For the same datasets as Step 2, we constructed another prediction model using randomly selected genes that are same number of the candidate set of DRGs in Step 2.

Step 4) We iterated Step 1 to 3 at 1000 times, thereby yielding 1000 pairs of AUCs between the candidate DRGs and randomly selected genes.

Step 5) Using a paired *t*-test, we compared the 1000 AUCs of a set of the interest DRGs with those of the randomly selected genes.

In Step 3, we selected random features to be compared or matched with the features from Step 2. For more appropriate comparison of the degree of predictive power between these sets, we randomly selected genes from different backgrounds. For example, in case of DEG_{AD} or DEG_{CVD}, the background genes for the random selection were all genes with a number of approximately 10,000. Besides, in case of DEG+eQTL, the background genes included the 5647 genes with eQTL evidence, and not all genes. From these processes, we generated different null distributions for the matched random gene set based on feature selection methods.

Cell type-specific DRGs from single cell RNA sequencing (For Section 2.7 in the main text)

We downloaded a heart CVD gene expression data (Accession ID: E-MTAB-7376) from a study by Farbehi and colleagues [15]. This study performed single-cell RNA sequencing at two times: one was for the total interstitial cell types (TIP, file name: *TIP_ShamVsMI_days3_7.txt*) and the other was for Pdgfra-GFP$^+$ fibroblast lineage cells (GFP, file name: *GFP_ShamVsMI _days3_7.txt*). In the TIP group, expression for 27999 transcripts and 15074 single cells of 24 cell types were measured by RNA-sequencing method, yielding a matrix consisting of 27999 rows and 17803 columns. In the GFP group, expression for 27999 transcripts and 17803 cells of 11 clusters were measured.

For each of all cell types, we established the mutually exclusive matrices for cells, yielding 24 and

11 gene expression matrices for TIP and GFP analyses, respectively. For each gene expression matrix, we removed transcripts (based on ENSEMBL ID) with bottom 30% of variance across samples, and remapped those to Entrez ID using 'useMart' function in biomaRt package [16]. Using 'DESeq' function in DESeq2 package [17], we measured gene level-fold change and their *P*-value between CVD (i.e., heart cells from MI operated mice) and control (i.e., heart cells from sham operated mice).

Comparison of the disease-related transcriptomic signature among blood and tissue gene expression datasets (For Section 3.3 in the main text)

We compared the phenotype-related transcriptomic signature between two tissues in the same disease or two diseases in the blood tissue by FC values for the selected genes between disease (AD or CVD) and matched control. For the large blood AD or CVD datasets (named as dataset 1), we measured the FC values for the selected genes that are one of eight combination cases in each disease between two conditions. Then, we calculated FC values for the selected genes between two statuses in other tissues or disease gene expression datasets (named as dataset 2). We measured Spearman correlation coefficient (SCC) between two lists of FC values of dataset 1 and dataset 2. We permuted label of the dataset 2 at 1000 times, and calculated FC values and their SCC with those of dataset 1, yielding a null distribution for the correlation values. We determined the top-rank of SCC between dataset 1 and 2 in the null distribution as the permuted *P*-value.

Measurement of overlap between two gene-sets (For Section 3.2 – 3.3 and Figure 3 in the main text)

We measured the degree of overlap between two gene sets using a hypergeometric test *p*-value. Considering two gene-sets G1 and G2, a *p*-value was computed by:

$$P\ \ value = \sum_{k=m}^{m} \frac{\binom{M}{m}\binom{N-M}{n-m}}{\binom{N}{n}}$$

where *N* represents the total number of genes in the gene expression dataset, *M* represents the number of genes in G1, *n* represents the number of genes in G2, and *m* represents the number of genes common between G1 and G2.

Figure S1. Comparison of disease-related signatures among the blood gene expression datasets. (A) Comparison of the FC values for all genes between two statuses (i.e., AD and CN) among 6 blood AD datasets. (B) Comparison of FC value for all genes between two statuses (e.g., CVD and CN) among 11 blood CVD datasets. AD, Alzheimer's disease; CVD, cardiovascular disease; CN, matched control.

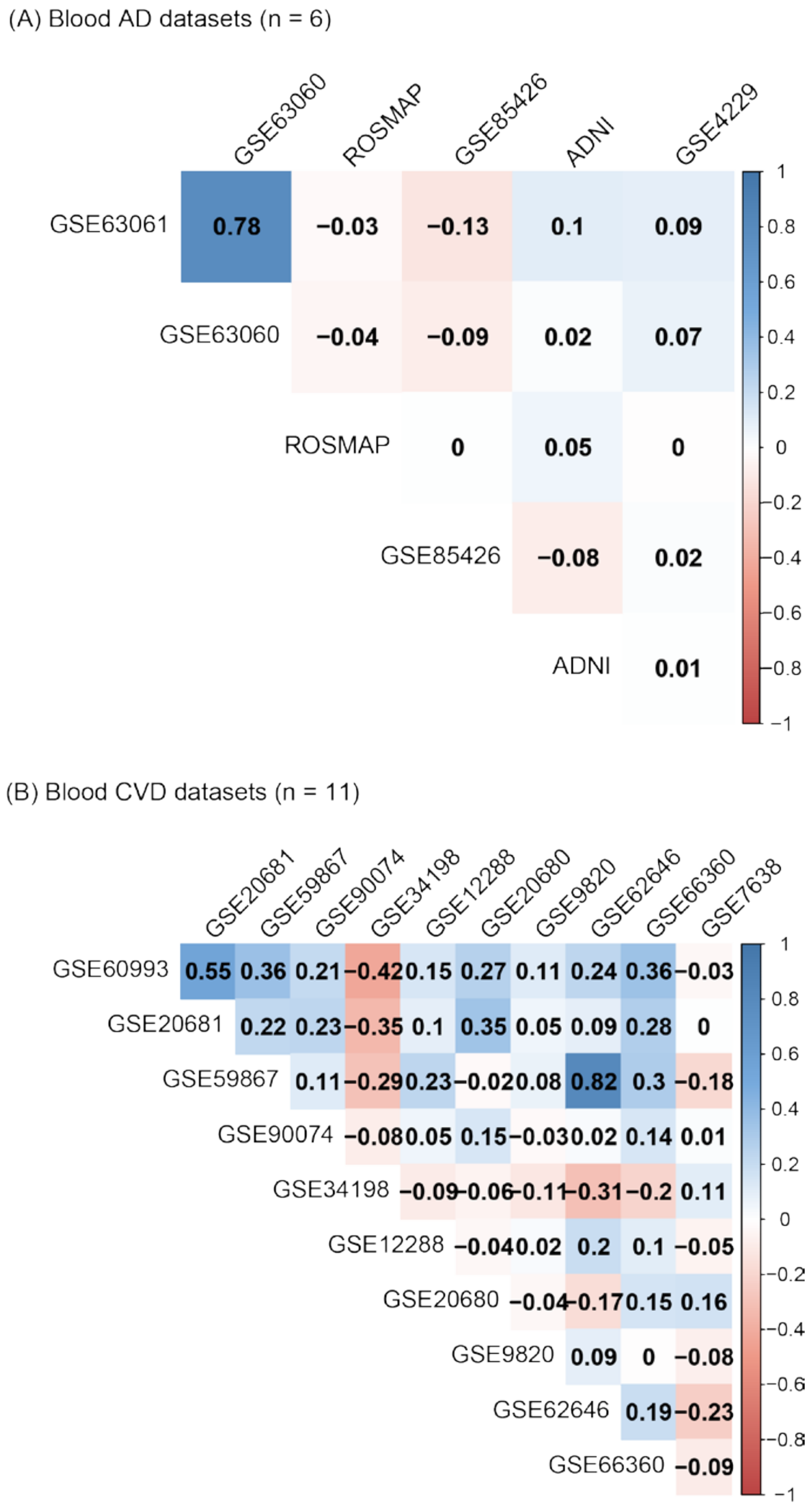

Figure S2. Comparison of disease-related transcriptomic signatures (fold changes between different conditions) for the selected AD-related genes obtained from the large blood AD dataset with those of the brain AD and blood CVD datasets. * denotes permuted *P*-value less than 0.05.

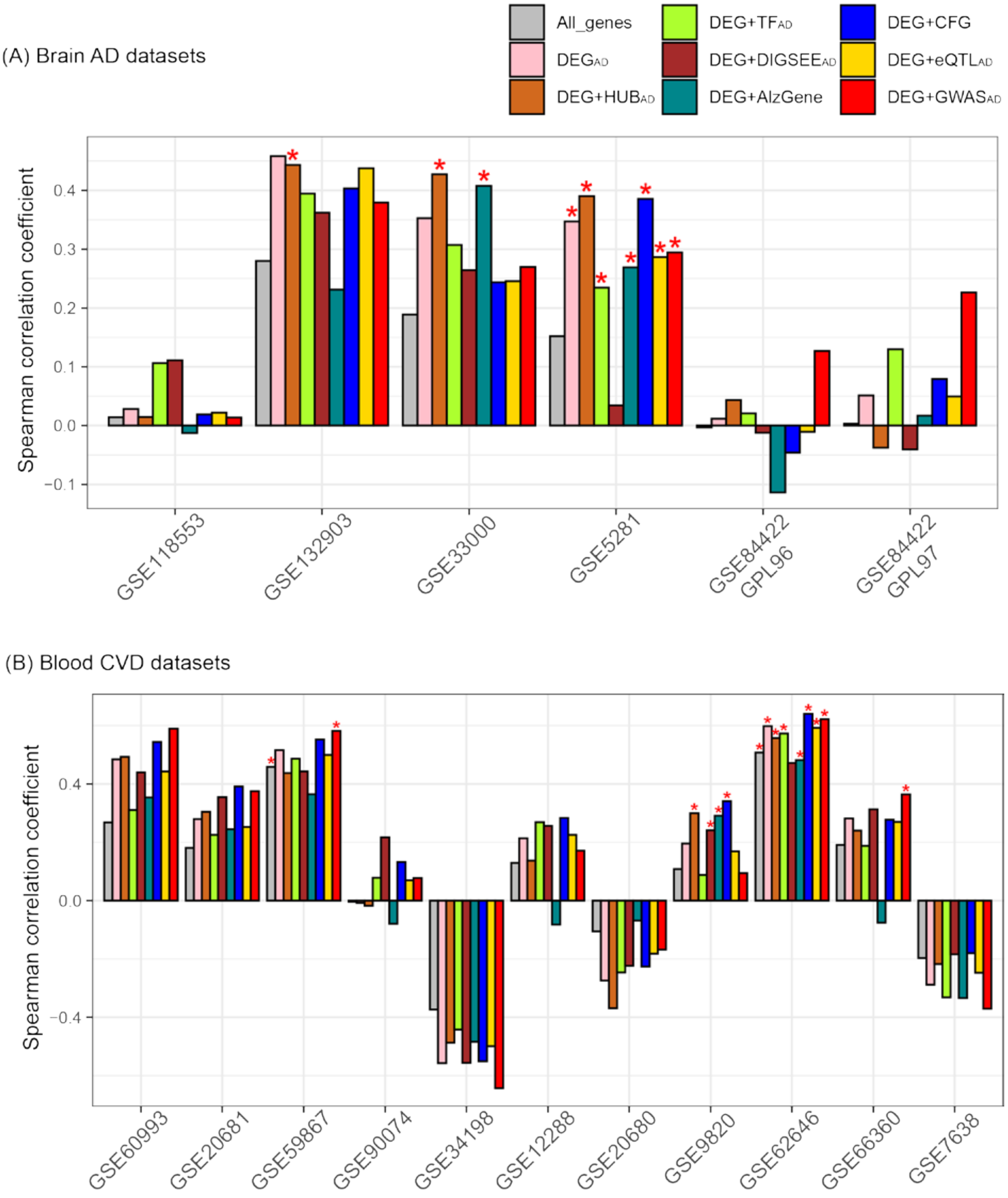

Figure S3. Comparison of disease-related transcriptomic signatures for the selected CVD-related genes obtained from the large blood CVD dataset with those of the tissue CVD and blood AD datasets. * denotes permuted *P*-value less than 0.05. GSE64566 is the super-series of GSE64554 (GSE64566 = GSE64554).

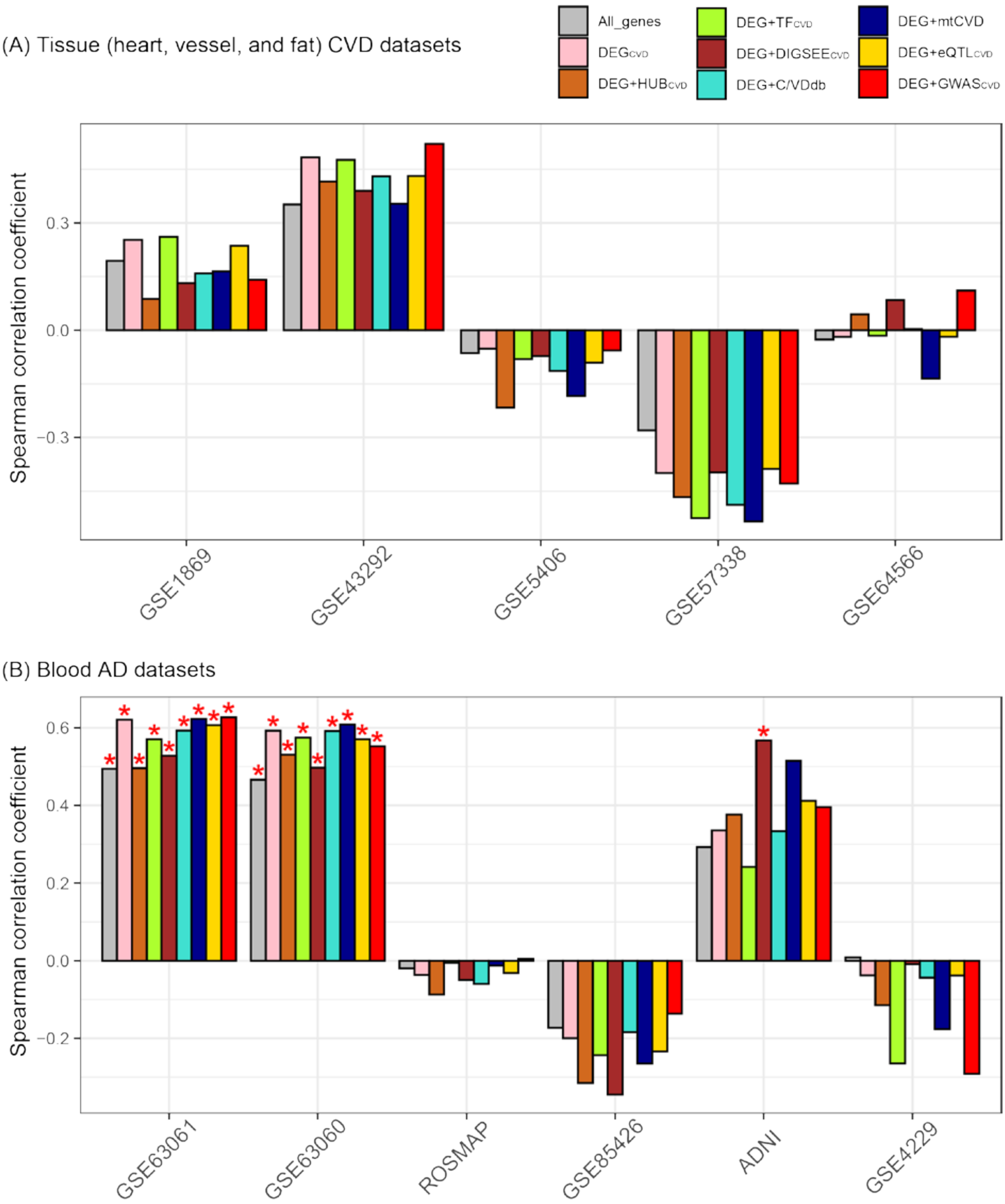

Figure S4. Performance of the eight blood AD-related gene sets for the prediction of brain AD.

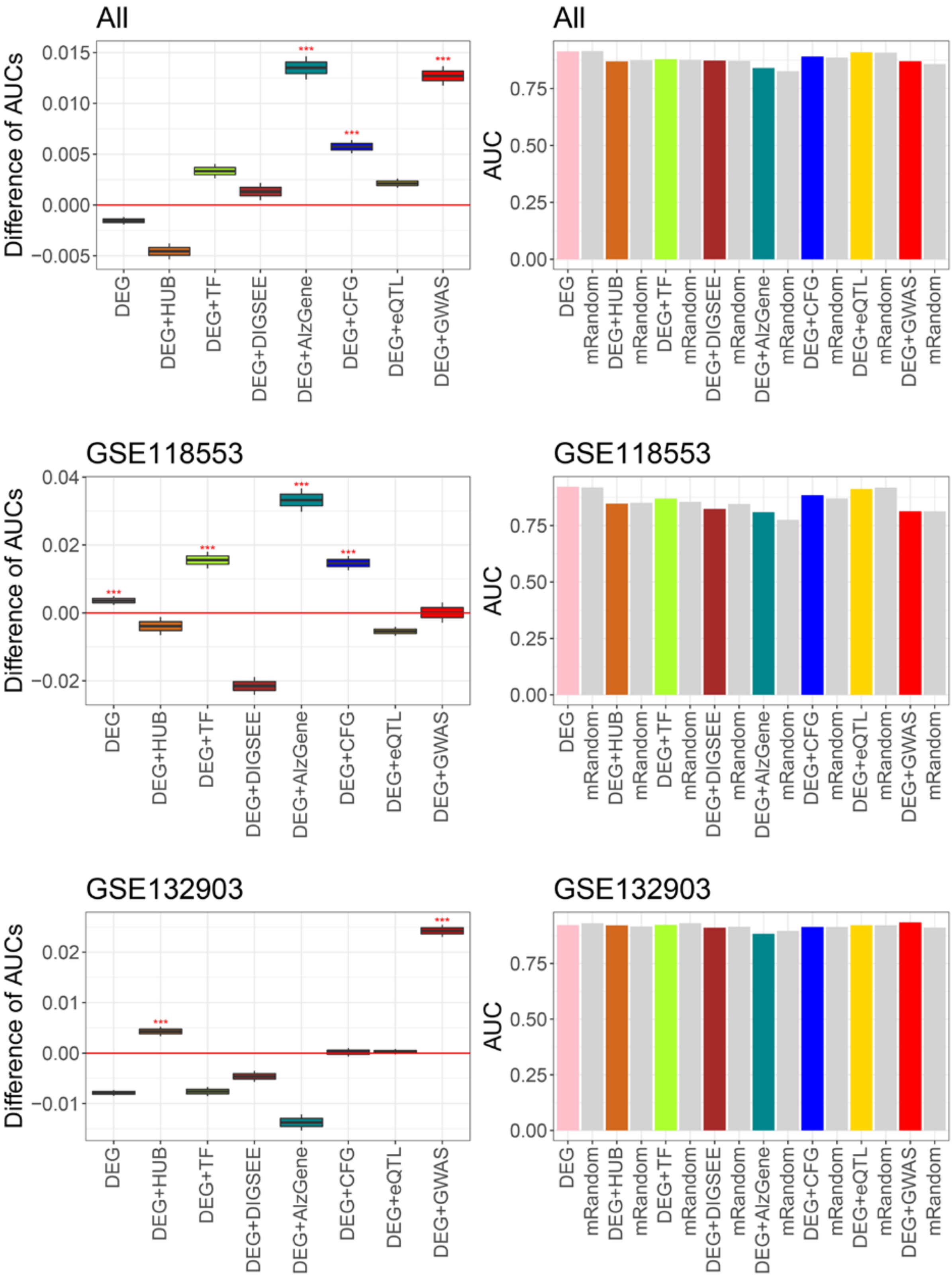

Figure S4. Performance of the eight blood AD-related gene sets for the prediction of brain AD (continued).

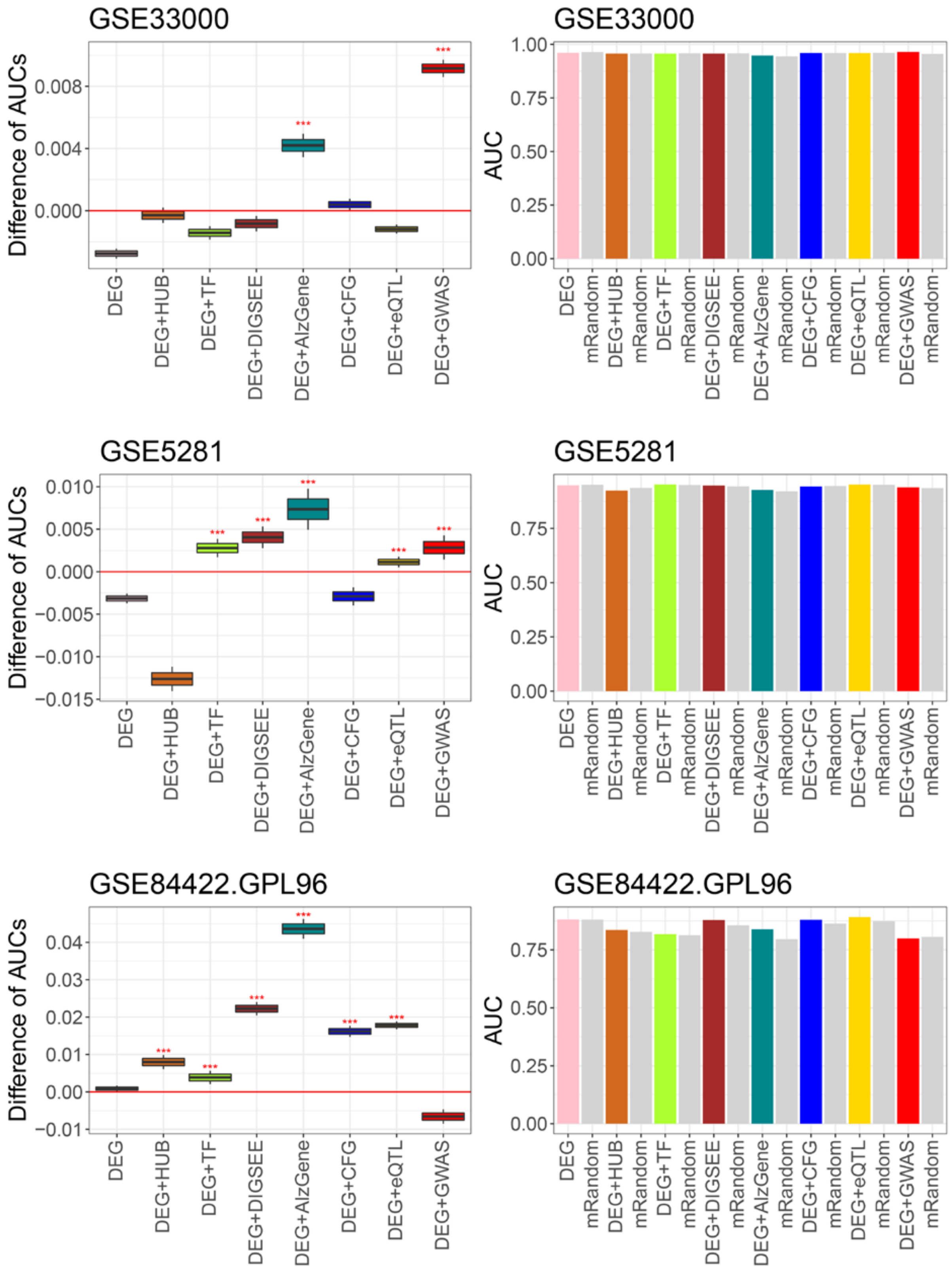

Figure S4. Performance of the eight blood AD-related gene sets for the prediction of brain AD (continued).

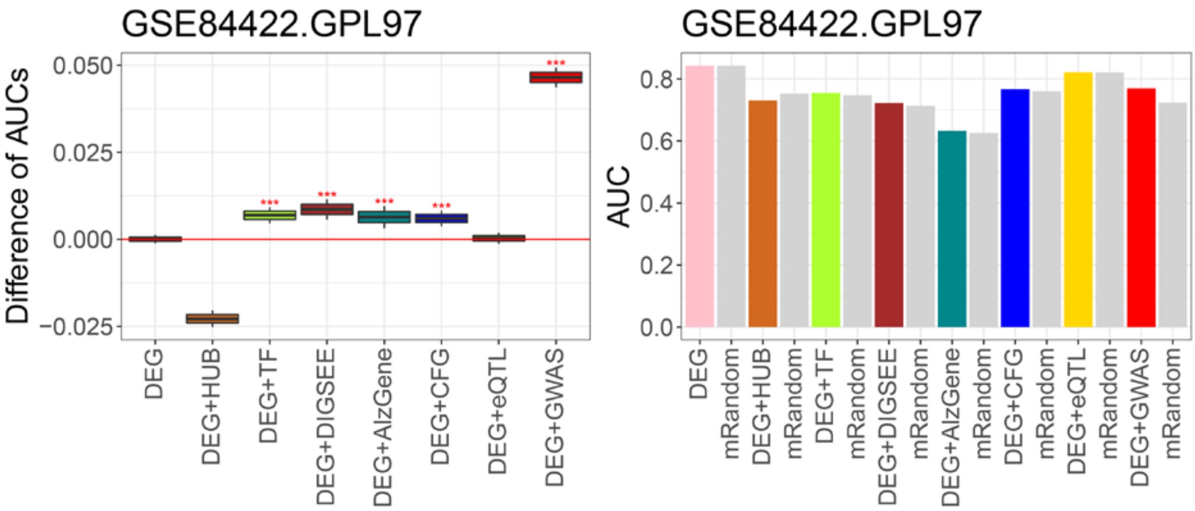

Figure S5. Performance of the eight blood AD-related gene sets for the prediction of blood CVD.

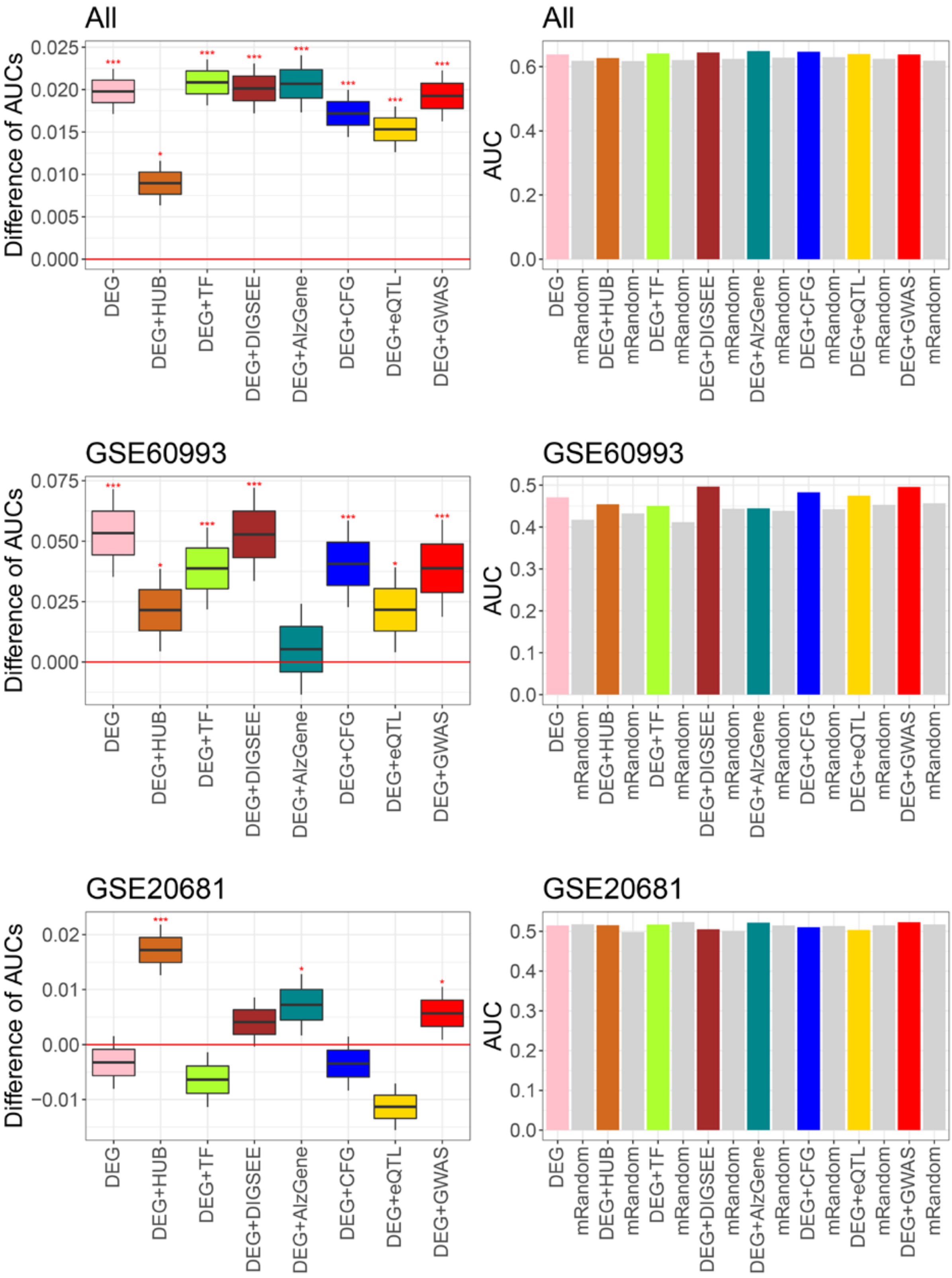

Figure S5. Performance of the eight blood AD-related gene sets for the prediction of blood CVD (continued).

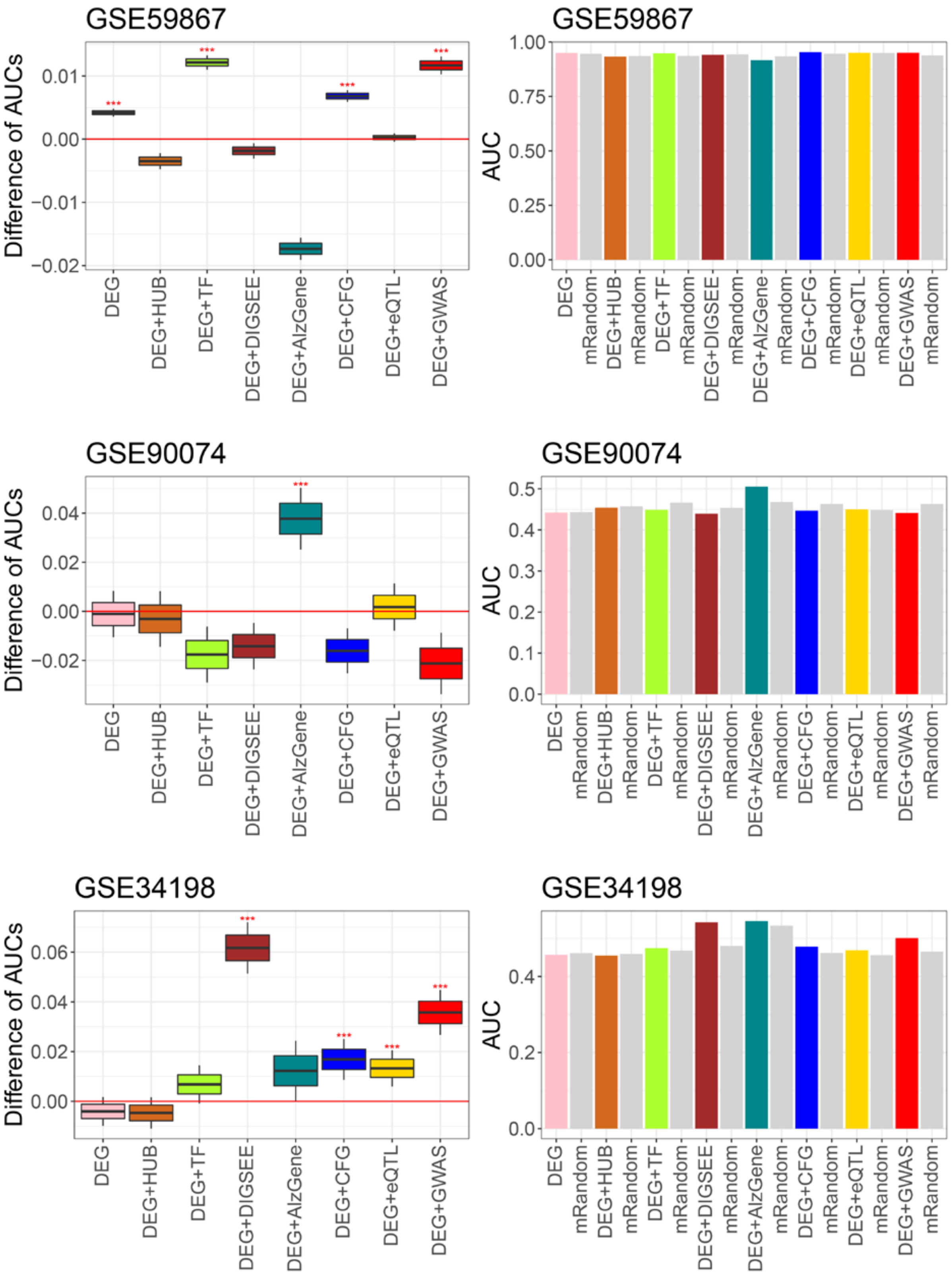

Figure S5. Performance of the eight blood AD-related gene sets for the prediction of blood CVD (continued).

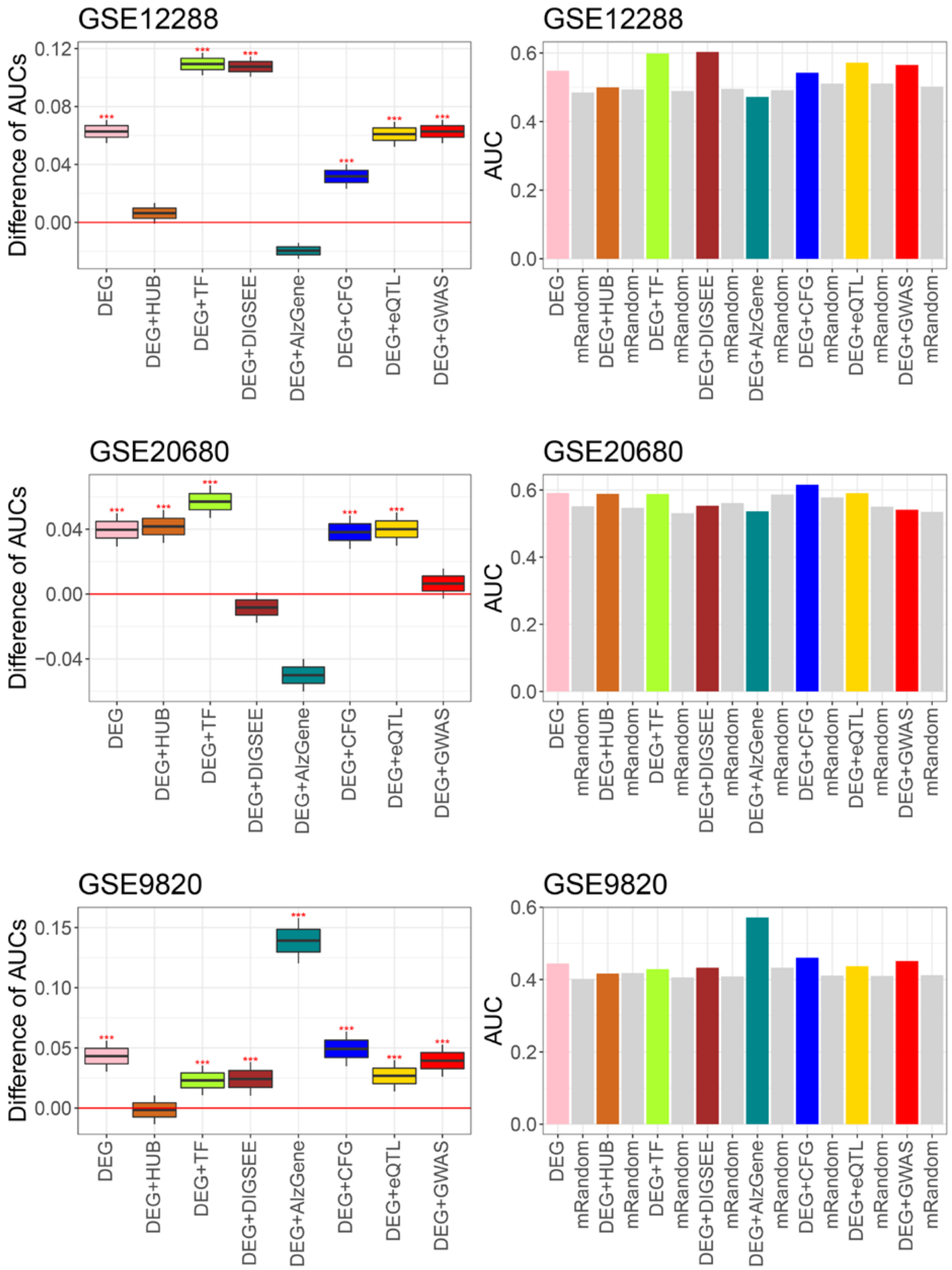

Figure S5. Performance of the eight blood AD-related gene sets for the prediction of blood CVD (continued).

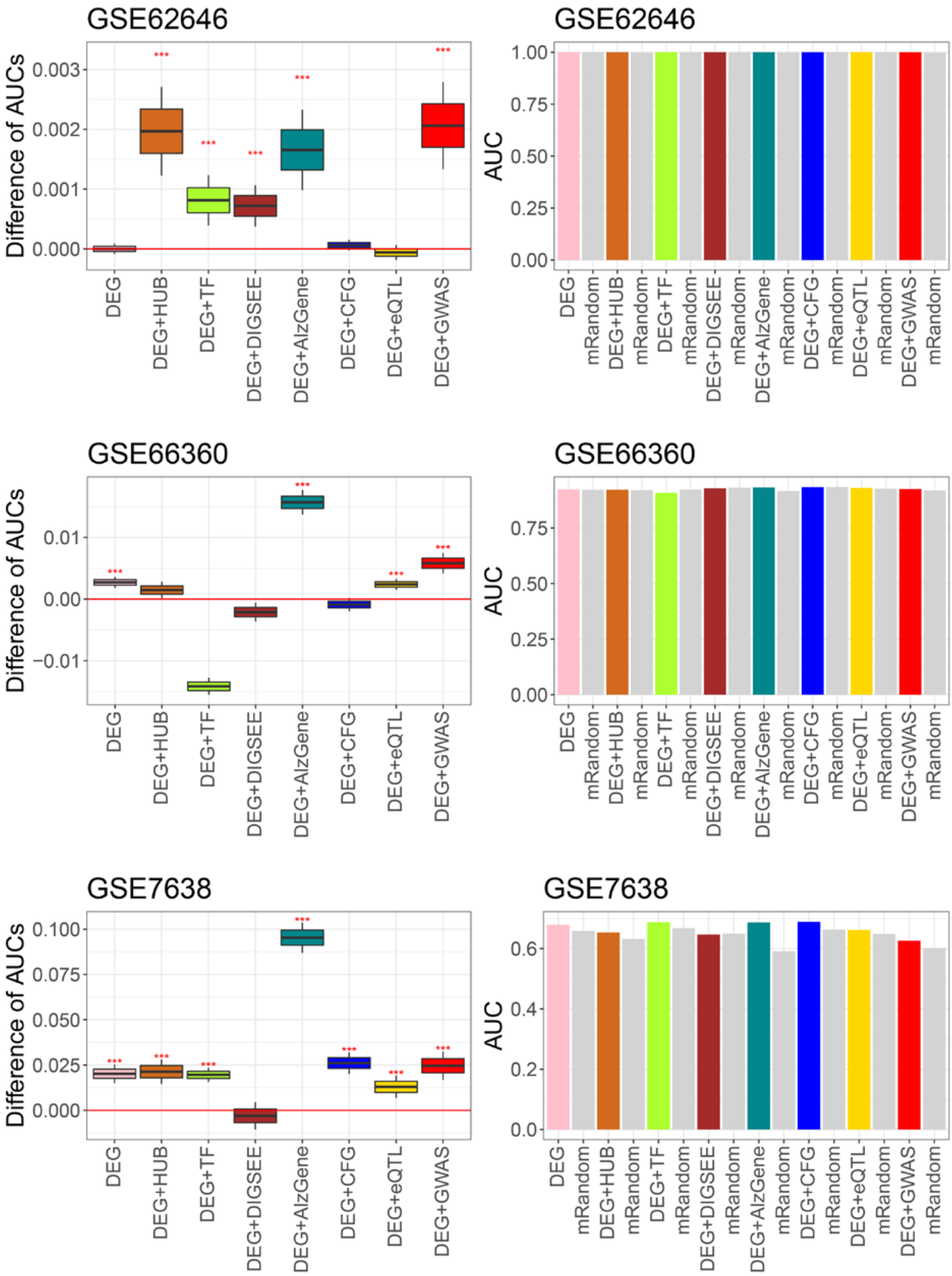

Figure S6. Performance of the eight blood CVD-related gene sets for the prediction of CVD tissues (heart, vessel, and fat) CVD. GSE64566 is the super-series of GSE64554 (GSE64566 = GSE64554).

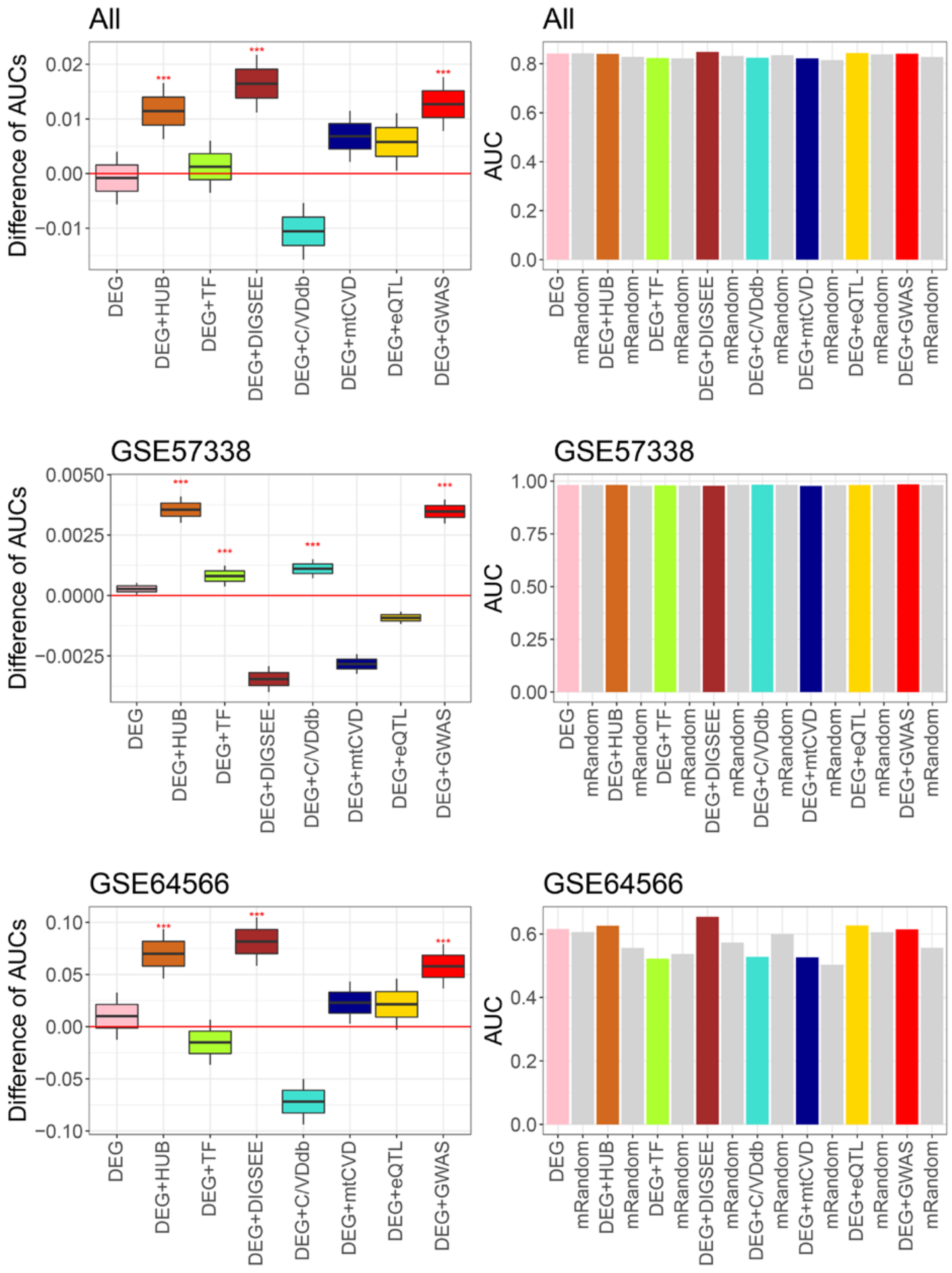

Figure S6. Performance of the eight blood CVD-related gene sets for the prediction of CVD tissues (heart, vessel, and fat) CVD (continued).

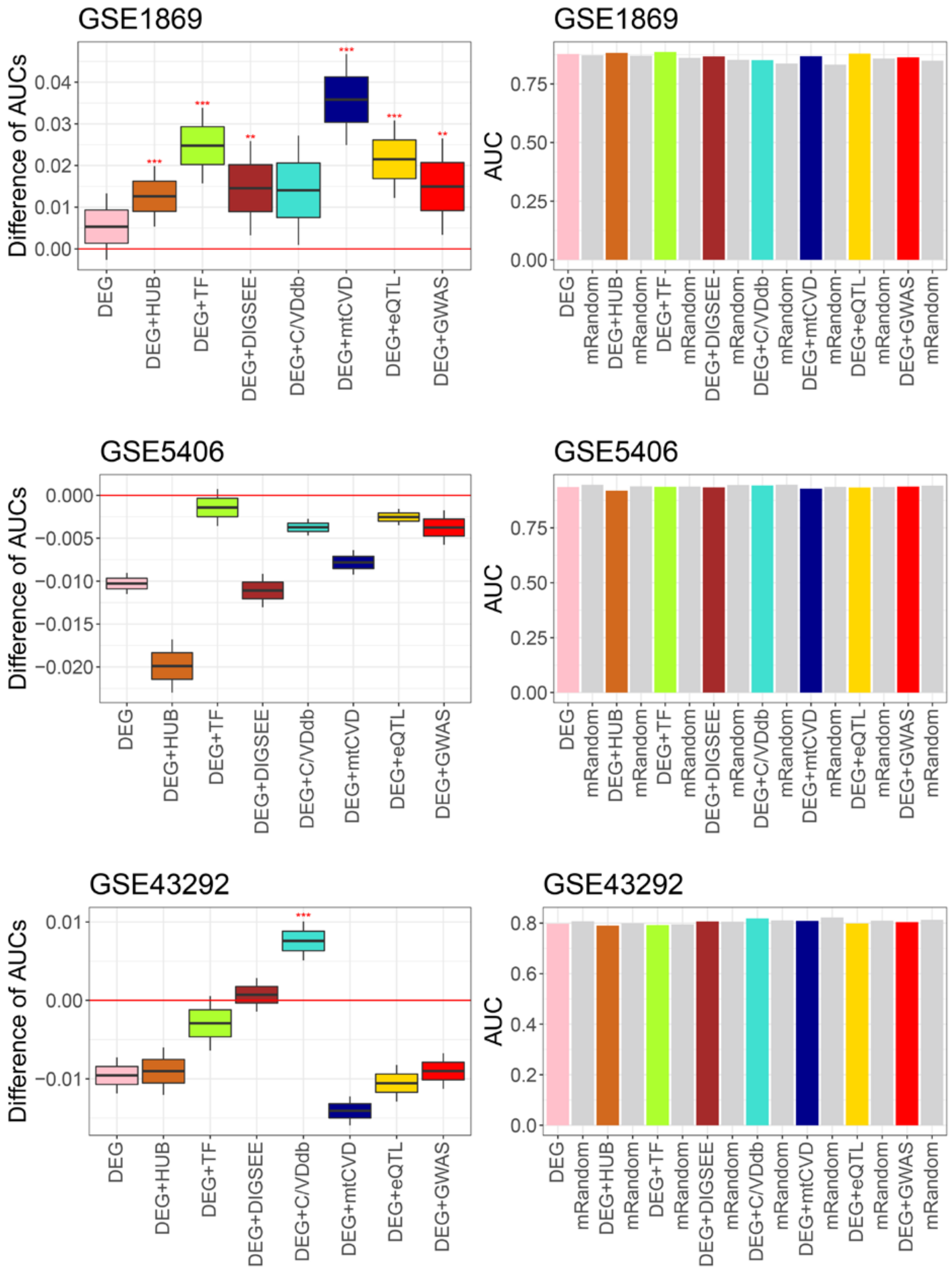

Figure S7. Performance of the eight blood CVD-related gene sets for the prediction of blood AD.

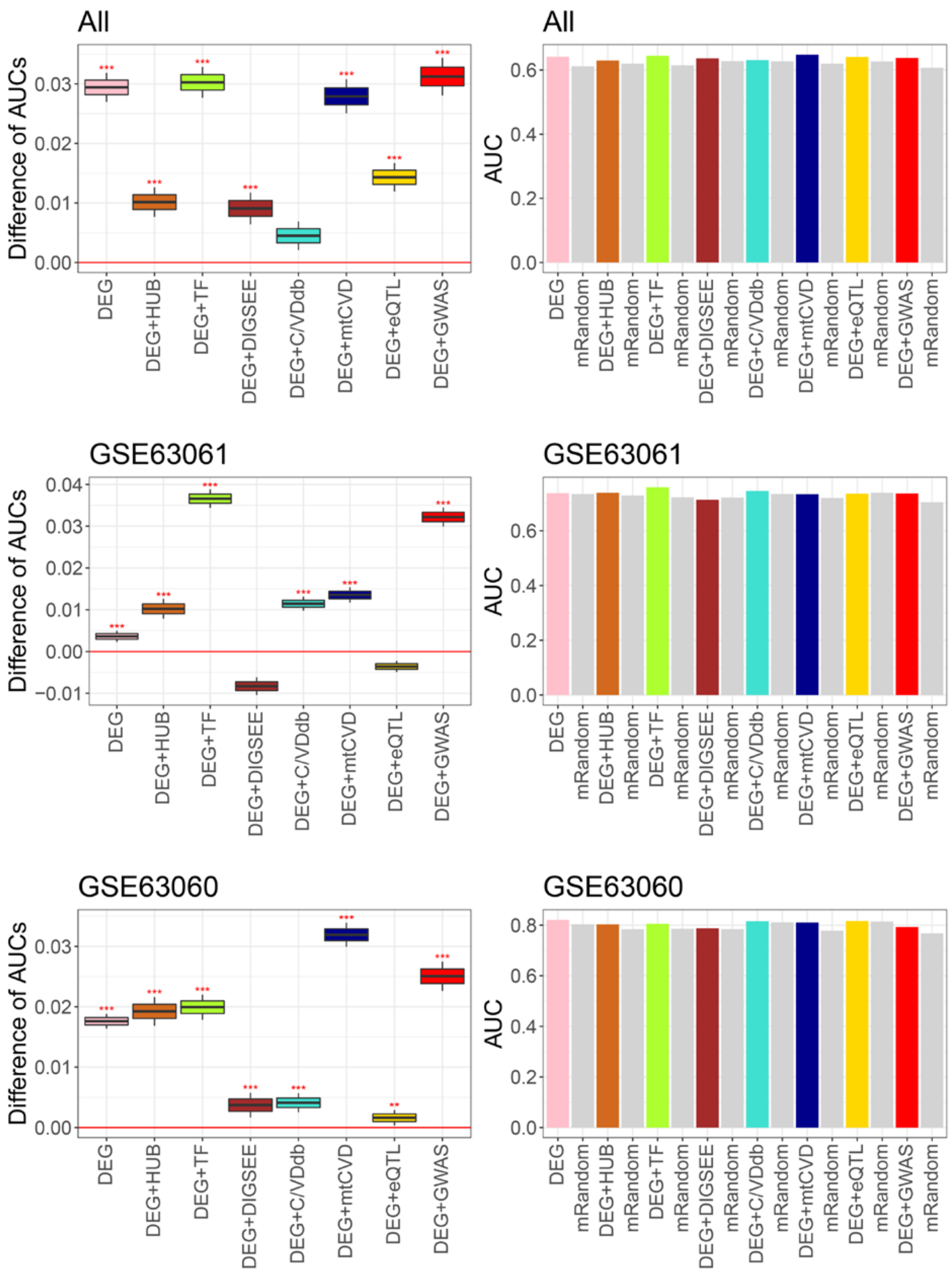

Figure S7. Performance of the eight blood CVD-related gene sets for the prediction of blood AD (continued).

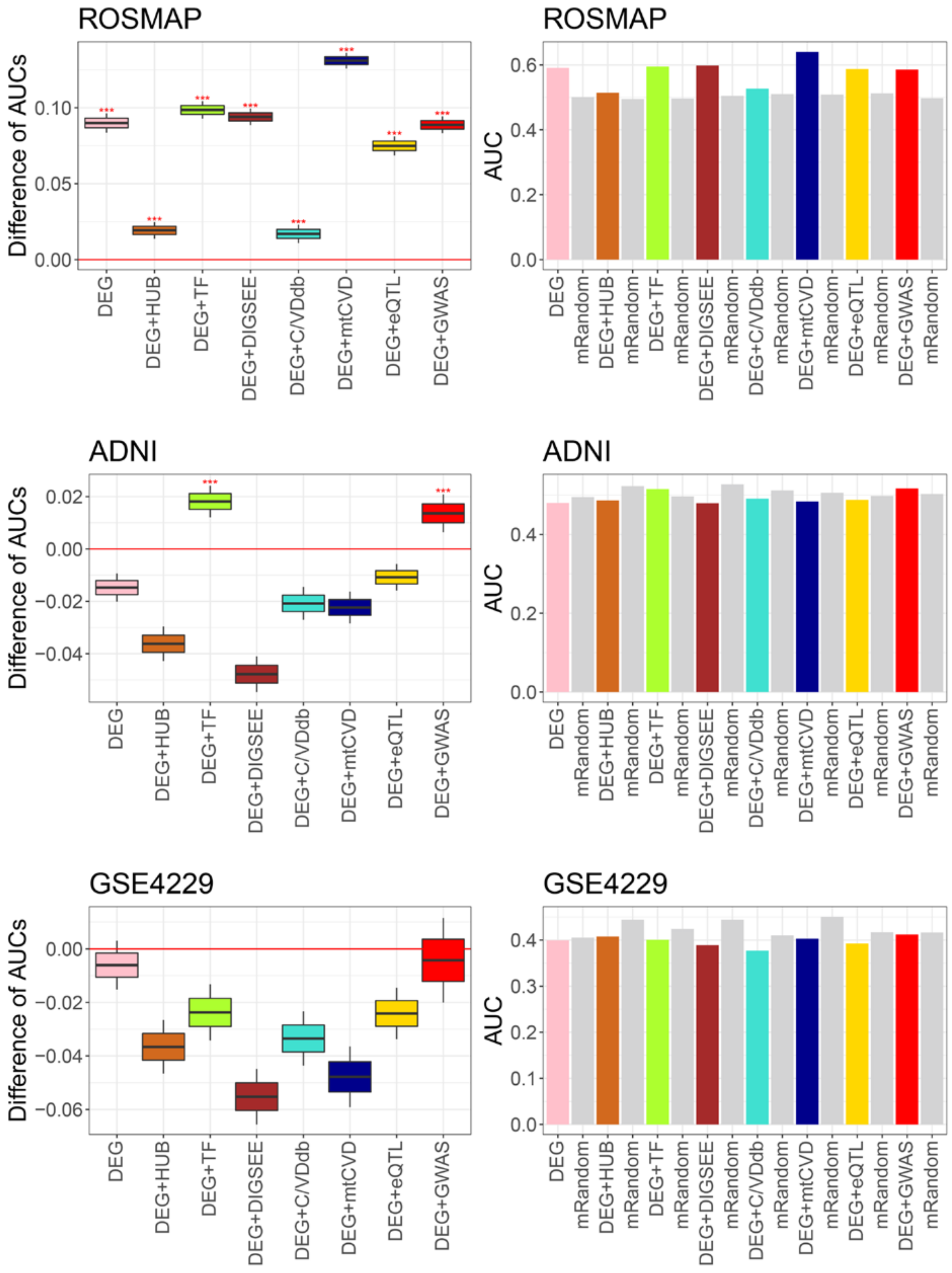

Figure S7. Performance of the eight blood CVD-related gene sets for the prediction of blood AD (continued).

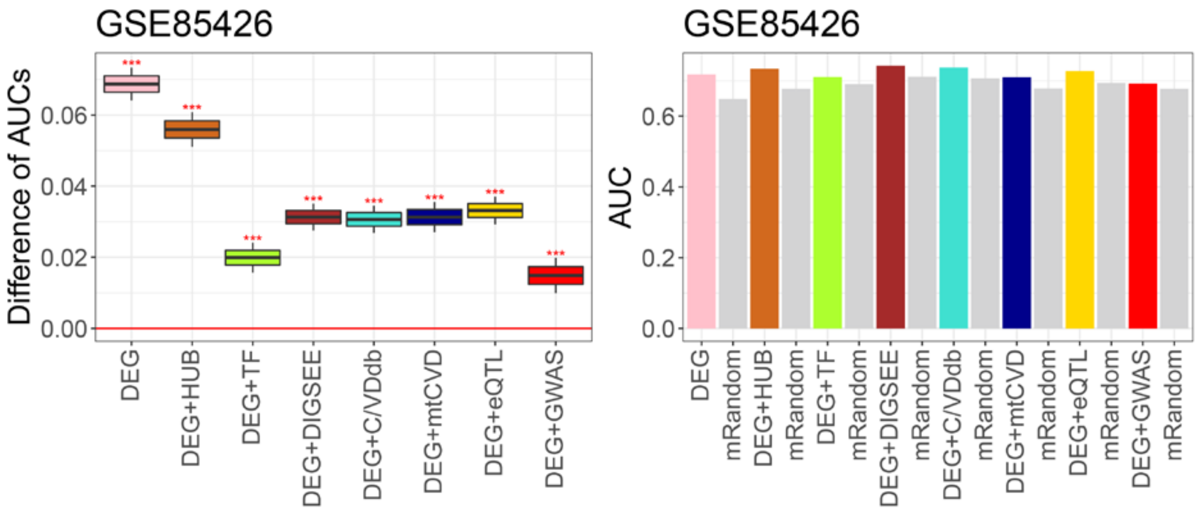

Supplementary Table S1. Genes in DEG+CFG

Entrez ID	Symbol
6166, 51258, 6230, 6233, 6119, 6229, 10312, 51373, 10987, 5052, 6224, 6727, 9535, 6170, 1622, 5685, 9931, 84233, 10347, 5689, 9168, 1933, 127018, 5356, 6206, 8569, 6160, 4830, 10671, 79716, 54849, 9166, 6165, 9978, 2799, 689, 6232, 4724, 5204, 37, 11224, 23166, 7297, 3684, 4697, 56945, 3678, 4116, 6156, 338692, 5706, 5695, 9181, 4704, 10287, 29927, 131076, 5901, 51522, 2961, 6667, 10904, 1441, 10213, 80279, 285381, 51380, 10307, 7411, 7316, 10938, 3101, 896, 126014, 83852, 51065, 2242, 54820, 23315, 51138, 51604, 5791, 23075, 84268, 87, 114790, 1520, 929, 7454, 4792, 581, 1653, 1051, 3455, 4722, 5034, 51690, 23228, 6548, 56647, 834, 2011, 2664, 7360, 9600, 6146, 81, 7414, 10577, 162989, 1497, 3308, 11180, 391, 1639, 55738, 3425, 7324, 51547, 5451, 6904, 1981, 23604, 5694, 2098, 51013, 5770, 10694, 79746, 64132, 7378, 6016, 10197, 3312, 56882, 3066, 23633, 5702, 196883, 3059, 5265, 488, 392, 6774, 23480, 6653, 1915, 4927, 7431, 23623, 4729, 7385, 10376, 6208, 4987, 7846, 988, 55052, 1471, 26054, 8766, 3423, 9056, 3636, 10507, 9709, 3329, 2720, 65056, 161, 23102, 7132, 1495, 5971, 80254, 1762, 5423, 57122, 5898, 1509, 4043, 4926, 80331, 7097, 5686, 6210, 25865, 818, 7535, 158, 3931, 2771, 998, 7332, 6041, 7076, 3281, 5579, 27141, 50808, 56271, 5495, 219931, 25796, 8460, 11059, 3936, 9135, 2051, 5293, 8726, 9869, 201475, 80709, 10001, 5889, 308, 55039, 6184, 1201, 6605, 217, 2791, 5986, 4299, 5329, 27072, 56987, 10910, 55907, 535, 2022, 974, 1429, 5800, 9424, 8273, 115992, 84128, 6583, 9394, 6850, 9112, 3796, 57231, 6195, 3157, 132, 1513, 3920, 9146, 112616, 6688, 5781, 4478, 2357, 2185, 55746, 84885, 103, 55343, 4176, 1831, 6281, 3148, 113675, 115817, 5336, 5501, 8312, 1043, 8878, 10111, 908, 5590, 152007	RPL36AL, MRPL51, RPS25, RPS27A, RPA3, RPS24, TCIRG1, MRPS17, COPS5, PRDX1, RPS20, SRP14, GMFG, RPL39, DBI, PSMA4, HELZ, TMEM126A, ABCA7, PSMB1, TMSB10, EEF1B2, LYPLAL1, PLRG1, RPS12, MKNK1, RPL31, NME1, DCTN6, NPEPL1, DEF8, EBAG9, RPL35A, RBX1, GNS, BTF3, RPS27, NDUFS4, PFDN5, ACADVL, RPL35, STAB1, TYK2, ITGAM, NDUFA4, MRPS22, ITGA5, MAGOH, RPL30, ANKRD13D, PSMC6, PSMB7, ARHGEF2, NDUFA9, RGS19, SEC61A1, CCDC58, RAN, TMEM14C, GTF2E2, SP1, BLCAP, CSF3R, PSMD14, CDK5RAP3, DPH3, CSAD, APBB3, VBP1, UBC, EHD1, HK3, CCND3, OSCAR, SETDB2, RPS27L, FES, NDE1, SLC9A8, COPS4, PIGT, PTPRE, SWAP70, RPAIN, ACTN1, STK11IP, CTSS, CD14, WAS, NFKBIA, BAX, DDX1, CEBPB, IFNAR2, NDUFS3, P4HB, LSM7, PLCL2, SLC9A1, BCCIP, CASP1, MARK2, GDI1, UGP2, PITPNM1, RPL22, ACTN4, VCL, NPC2, DEDD2, CTNS, HSPA4, WDR6, RHOG, DCTN1, ARFGAP1, IDUA, UBE2E1, SIRT7, POU2F1, TBCD, EIF4G1, DAPK2, PSMB6, ESD, EXOSC1, PTPN1, CCT8, ECHDC3, XYLT2, UPP1, RIT1, PSME3, HSPA8, CDC42SE1, HDAC2, KPNA6, PSMC3, ADCY4, HCLS1, SERPINA1, ATP2A2, ARHGAP1, STAT3, SEC61G, SORL1, EEF1A1, NUP88, VIM, RUSC1, NDUFV2, UQCRC2, TUBA1B, RPS14, OPRL1, TUBA1A, CDC5L, MRPL20, CST3, SENP6, RAB11A, IDS, SLC7A7, INPPL1, SEMA4D, HERPUD1, HSPD1, GLB1, GPBP1, AP2A2, TBC1D2B, TNFRSF1A, CTNNA1, RELB, CEP63, DMWD, POLB, NUP107, RALA, CTSD, LRPAP1, NUMA1, DNAJC5, TLR2, PSMA5, RPS15A, PRKD2, CAMK2G, ZAP70, ADSL, LCAT, GNAI2, CDC42, UBE2L3, RNASEL, TIMP1, HSBP1, PRKCB, CIDEB, AK3, BEX4, PPM1B, TPCN2, PGLS, TPST1, WWP1, LCP1, RABEP1, EPHB6, PIK3CD, EED, SETDB1, RAB12, AKNA, MED6, RAD51C, ANXA5, TRMT12, RPN1, CLN3, SMARCE1, ALDH2, GNG11, RFNG, AFF1, PLAUR, VPS41, BBX, SUGT1, CMAS, ATP6V0A1, ENG, CD79B, CRYZ, PTPRO, KCNK6, SLC10A3, RNF166, WDR75, SLC22A4, HS6ST1, SYK, MTA1, KIF2A, SNX14, RPS6KA1, HMGCS1, ADK, CTSK, LAMP2, HGS, CMTM7, SPI1, PTPN11, MSN, FPR1, PTK2B, NUP133, ZDHHC12, ADAR, SLC35C1, MCM7, TSC22D3, S100A10, HMGB2, SDSL, DHRS1, PLCG2, PPP1CC, AXIN1, CD52, SQSTM1, RAD50, CCT6A, PRKCZ, GLIPR2

Supplementary Table S2. Genes in DEG+DIGSEE$_{CVD}$

Entrez ID	Symbol
26301, 706, 366, 1312, 9935, 6280, 929, 4043, 3676, 902, 581, 240, 272, 945, 3684, 134957, 23198, 472, 1535, 5562, 3824, 10460, 133, 55902, 2907, 912, 6850, 1378, 5595, 968, 1891, 11188, 9360, 4358, 10226, 2678, 1052, 535, 682, 841, 9158, 5351, 6775, 7077, 23765, 197131, 22914, 3084, 55968, 1509, 5724, 7402, 54472, 2597, 27239, 4068, 2319, 10437, 1230, 5055, 602, 5265, 604, 2357, 3557, 754, 6520, 23047, 26191, 6095, 2153, 115361, 2210, 3636, 64127, 6279, 2217, 4815, 27241, 23569, 1471, 4210, 79147, 55819, 2212, 949, 5467, 463, 7056, 2534, 4683, 3303, 9332, 51548, 59343, 10221, 23345, 27347, 7138, 1593, 545, 6558, 10219, 10482, 1066, 728, 6256, 5290, 10666, 8673, 7132, 4615, 6548, 7099, 5696, 4609, 65056, 3956, 1439, 1978, 9535, 4814, 4482, 353, 1241, 4607, 9842, 8877, 330, 967, 7421, 1890, 5329, 6584, 7840, 55669, 4688, 55210, 3570, 55850, 6772, 5583, 376497, 943, 10008, 5295, 1629, 6464, 7076, 572, 6901, 177, 54210, 8065, 116985, 9123, 4524, 1827, 7408, 948, 7852, 22921, 80271, 6515, 5580, 6197, 5310, 217, 58488, 4691, 51070, 1340, 10397, 79602, 1026, 262, 10133, 2876, 3732, 1889, 302, 5294, 10333, 8320, 847, 57142, 6272, 3784, 5091, 4056, 118429, 6778, 2355, 2885, 1434, 8728, 83852, 7411, 9927, 8834, 396, 977, 283, 2950, 23327, 3992, 3315, 1436, 719, 9049, 23082, 56998, 10519, 23586, 490, 6382, 3689	GBGT1, TSPO, AQP9, COMT, MAFB, S100A9, CD14, LRPAP1, ITGA4, CCNH, BAX, ALOX5, AMPD3, CD33, ITGAM, STXBP5, PSME4, ATM, CYBA, PRKAA1, KLRD1, TACC3, ADM, ACSS2, GRINA, CD1D, SYK, CR1, MAPK3, CD68, ECH1, NISCH, PPIG, MPV17, PLIN3, GGT1, CEBPD, ATP6V0A1, BSG, CASP8, FIBP, PLOD1, STAT4, TIMP2, IL17RA, UBR1, KLRK1, NRG1, NSFL1C, CTSD, PTAFR, UTRN, TOLLIP, GAPDH, GPR162, SH2D1A, FLOT2, IFI30, CCR1, SERPINB2, BCL3, SERPINA1, BCL6, FPR1, IL1RN, PTTG1IP, SLC3A2, PDS5B, PTPN22, RORA, F5, GBP4, FCGR1B, INPPL1, NOD2, S100A8, FCGRT, NINJ2, BBS9, PADI4, CST3, MEFV, FKRP, RNF130, FCGR2A, SCARB1, PPARD, ZFHX3, THBD, FYN, NBN, HSPA1A, CD163, SIRT6, SENP2, TRIB1, SYNE1, STK39, TNNT1, CYP27A1, ATR, SLC12A2, KLRG1, NXF1, CES1, C5AR1, RXRA, PIK3CA, CD226, VAMP8, TNFRSF1A, MYD88, SLC9A1, TLR4, PSMB8, MYC, GPBP1, LGALS1, CSF2RB, EIF4EBP1, GMFG, NINJ1, MSRA, APRT, LTB4R, MYBPC3, PLEKHM1, SPHK1, BIRC3, CD63, VDR, TYMP, PLAUR, SLC22A5, ALMS1, MFN1, NCF2, ATAD3A, IL6R, USE1, STAT1, PRKCH, SLC27A1, TNFRSF8, KCNE3, PIK3R1, DBT, SHC1, TIMP1, BAD, TAZ, AGER, TREM1, CUL5, ARAP1, SLC16A3, MTHFR, RCAN1, VASP, CD36, CXCR4, MSRB2, ITPKC, SLC2A3, PRKCD, RPS6KA3, PKD1, ALDH2, PCTP, NCL, NOSIP, COX6B1, NDRG1, ADIPOR2, CDKN1A, AMD1, OPTN, GPX1, CD82, ECE1, ANXA2, PIK3CG, TLR6, EOMES, CAT, RTN4, SORT1, KCNQ1, PC, LTC4S, ANTXR2, STAT6, FOSL2, GRB2, CSE1L, ADAM19, SETDB2, VBP1, MFN2, TMEM11, ARHGDIA, CD151, ANG, GSTP1, NEDD4L, FADS1, HSPB1, CSF1R, C3AR1, AIP, PPRC1, CTNNBIP1, CIB1, DDX58, ATP2B1, SDC1, ITGB2

Supplementary Table S3. Parent genes with a significantly changed number of child genes in disease gene regulatory network.

Parent genes in AD-GRN	Parent genes in CVD-GRN
GPBP1, STAT3,<br>ADAR, HDAC2,<br>CDK5RAP3, AKNA,<br>PRKCB, EED,<br>POU2F1, PRKD2,<br>HMGB2, PRKCZ,<br>HSBP1, SETDB2,<br>SETDB1, SP1,<br>RELB, APBB3,<br>HELZ, MTA1	SETDB2, TLR4,<br>ATR, CIB1,<br>MSRB2, STAT4,<br>PDS5B, CEBPD,<br>GPBP1, MAFB,<br>ITGB2, CTNNBIP1,<br>BCL6, CDKN1A,<br>TRIB1, CD36,<br>ATM, SYK,<br>HSPA1A, PPARD,<br>VDR, S100A9,<br>RCAN1, CAT,<br>MYD88

References

1. Clough, E. and T. Barrett, *The Gene Expression Omnibus Database.* Methods Mol Biol, 2016. **1418**: p. 93-110.
2. Bennett, D.A., et al., *Overview and findings from the religious orders study.* Curr Alzheimer Res, 2012. **9**(6): p. 628-45.
3. Greenwood, A.K., et al., *The AD Knowledge Portal: A Repository for Multi-Omic Data on Alzheimer's Disease and Aging.* Curr Protoc Hum Genet, 2020. **108**(1): p. e105.
4. Mostafavi, S., et al., *A molecular network of the aging human brain provides insights into the pathology and cognitive decline of Alzheimer's disease.* Nat Neurosci, 2018. **21**(6): p. 811-819.
5. Dobin, A., et al., *STAR: ultrafast universal RNA-seq aligner.* Bioinformatics, 2013. **29**(1): p. 15-21.
6. Li, B. and C.N. Dewey, *RSEM: accurate transcript quantification from RNA-Seq data with or without a reference genome.* BMC bioinformatics, 2011. **12**(1): p. 1-16.
7. Johnson, W.E., C. Li, and A. Rabinovic, *Adjusting batch effects in microarray expression data using empirical Bayes methods.* Biostatistics, 2007. **8**(1): p. 118-127.
8. Irizarry, R.A., et al., *Exploration, normalization, and summaries of high density oligonucleotide array probe level data.* Biostatistics, 2003. **4**(2): p. 249-264.
9. Lee, T. and H. Lee, *Prediction of Alzheimer's disease using blood gene expression data.* Sci Rep, 2020. **10**(1): p. 3485.
10. Kittleson, M.M., et al., *Gene expression analysis of ischemic and nonischemic cardiomyopathy: shared and distinct genes in the development of heart failure.* Physiol Genomics, 2005. **21**(3): p. 299-307.
11. Hannenhalli, S., et al., *Transcriptional genomics associates FOX transcription factors with human heart failure.* Circulation, 2006. **114**(12): p. 1269-76.
12. Liu, Y., et al., *RNA-Seq identifies novel myocardial gene expression signatures of heart failure.* Genomics, 2015. **105**(2): p. 83-9.
13. Vacca, M., et al., *Integrative miRNA and whole-genome analyses of epicardial adipose tissue in patients with coronary atherosclerosis.* Cardiovasc Res, 2016. **109**(2): p. 228-39.
14. Langfelder, P. and S. Horvath, *WGCNA: an R package for weighted correlation network analysis.* BMC bioinformatics, 2008. **9**(1): p. 559.
15. Farbehi, N., et al., *Single-cell expression profiling reveals dynamic flux of cardiac stromal, vascular and immune cells in health and injury.* Elife, 2019. **8**.

16. Durinck, S., et al., *BioMart and Bioconductor: a powerful link between biological databases and microarray data analysis.* Bioinformatics, 2005. **21**(16): p. 3439-3440.
17. Love, M.I., W. Huber, and S. Anders, *Moderated estimation of fold change and dispersion for RNA-seq data with DESeq2.* Genome biology, 2014. **15**(12): p. 1-21.
